# BABA-induced pathogen resistance: a multi-omics analysis of the tomato response reveals a hyper-receptive status involving ethylene

**DOI:** 10.1093/hr/uhad068

**Published:** 2023-04-13

**Authors:** Martina Zapletalová, Corinne Rancurel, Benoit Industri, Marc Bardin, Kevin Le Brigand, Philippe Nicot, Virginie Magnone, Aurélie Seassau, Pascal Barbry, David Potěšil, Zbyněk Zdráhal, Michel Ponchet, Jan Lochman

**Affiliations:** UMR Institut Sophia Agrobiotech INRAE 1355, CNRS 6254, Université Côte d’Azur, 400, Route des Chappes, 06903 Sophia Antipolis, France; Department of Biochemistry, Faculty of Science, Masaryk University, Kotlářská 2, 61137 Brno, Czech Republic; UMR Institut Sophia Agrobiotech INRAE 1355, CNRS 6254, Université Côte d’Azur, 400, Route des Chappes, 06903 Sophia Antipolis, France; UMR Institut Sophia Agrobiotech INRAE 1355, CNRS 6254, Université Côte d’Azur, 400, Route des Chappes, 06903 Sophia Antipolis, France; Unité 407 Pathologie végétale, INRAE, Domaine Saint-Maurice, 84143 Montfavet cedex, France; UMR Institut de Pharmacologie Moléculaire et Cellulaire, Université Côte d’Azur, CNRS 7275, 660 Route des Lucioles 06560 Valbonne, France; Unité 407 Pathologie végétale, INRAE, Domaine Saint-Maurice, 84143 Montfavet cedex, France; UMR Institut de Pharmacologie Moléculaire et Cellulaire, Université Côte d’Azur, CNRS 7275, 660 Route des Lucioles 06560 Valbonne, France; UMR Institut Sophia Agrobiotech INRAE 1355, CNRS 6254, Université Côte d’Azur, 400, Route des Chappes, 06903 Sophia Antipolis, France; UMR Institut de Pharmacologie Moléculaire et Cellulaire, Université Côte d’Azur, CNRS 7275, 660 Route des Lucioles 06560 Valbonne, France; Central European Institute of Technology, Masaryk University, Kamenice 5, 625 00 Brno, Czech Republic; Central European Institute of Technology, Masaryk University, Kamenice 5, 625 00 Brno, Czech Republic; UMR Institut Sophia Agrobiotech INRAE 1355, CNRS 6254, Université Côte d’Azur, 400, Route des Chappes, 06903 Sophia Antipolis, France; Department of Biochemistry, Faculty of Science, Masaryk University, Kotlářská 2, 61137 Brno, Czech Republic

## Abstract

Prior exposure to microbial-associated molecular patterns or specific chemical compounds can promote plants into a primed state with stronger defence responses. β-aminobutyric acid (BABA) is an endogenous stress metabolite that induces resistance protecting various plants towards diverse stresses. In this study, by integrating BABA-induced changes in selected metabolites with transcriptome and proteome data, we generated a global map of the molecular processes operating in BABA-induced resistance (BABA-IR) in tomato. BABA significantly restricts the growth of the pathogens *Oidium neolycopersici* and *Phytophthora parasitica* but not *Botrytis cinerea*. A cluster analysis of the upregulated processes showed that BABA acts mainly as a stress factor in tomato. The main factor distinguishing BABA-IR from other stress conditions was the extensive induction of signaling and perception machinery playing a key role in effective resistance against pathogens. Interestingly, the signalling processes and immune response activated during BABA-IR in tomato differed from those in *Arabidopsis* with substantial enrichment of genes associated with jasmonic acid (JA) and ethylene (ET) signalling and no change in Asp levels. Our results revealed key differences between the effect of BABA on tomato and other model plants studied until now. Surprisingly, salicylic acid (SA) is not involved in BABA downstream signalization whereas ET and JA play a crucial role.

## Introduction

Throughout their lives, plants are constantly exposed to many stressful situations caused by changing environmental conditions or attacks by various pests and pathogenic microorganisms. Therefore, they have evolved structural barriers, microbicidal secondary metabolites, and inducible defence mechanisms to repel potential attackers. Unfortunately, the basal immune responses of plants are usually only sufficient to slow their colonisation by pathogens. As a result, a significant portion of the world’s plant production is destroyed each year by fungi, oomycetes, bacteria, insects, and nematodes [1]. Although plants do not have the adaptive immune system of vertebrates, it has long been known that components of the innate immune system of plants can learn from the past [2]. When exposed to microbe-associated molecular patterns (MAMPs) or specific chemical compounds, plants can enter a state of enhanced defence characterised by more rapid and robust responses to stressful stimuli. Although the term “defence priming” was proposed in the 1930s, the molecular mechanisms underlying this phenomenon have only recently been partially elucidated, particularly in the model plant *Arabidopsis thaliana* [3, 4]. Defence priming causes increased expression of genes related to stress and defence [5], including many transcription factors that regulate defence [6], and is now considered an essential component of several types of systemic plant immunity, including acquired systemic acquired resistance (SAR), induced systemic resistance (ISR) [3, 7], wound-induced resistance [8], and resistance induced by chemical compounds. Unlike strategies based on single resistance genes, defence priming activates multigenic defence mechanisms, conferring relatively durable resistance [3]. One of the most effective priming agents is the non-protein amino acid β-aminobutyric acid (BABA), which protects various plant species against a wide range of stresses [9]. Importantly, this resistance is long-lasting and can be transferred to vegetative progeny [10, 11]. BABA was previously considered a xenobiotic, but it has recently been shown to accumulate in stress-exposed plants [12], suggesting that it is an endogenous stress metabolite [13]. BABA induces resistance via the action of several hormones, including salicylic acid (SA) [14, 15], jasmonic acid (JA) [16], abscisic acid (ABA) [17], and ethylene (ET) [15]; the signalling pathway that is activated appears to depend strongly on the particular plant-pathogen combination [6]. Recently, it was discovered that the aspartyl-tRNA synthetase (AspRS) IBI1 in *A. thaliana* serves as an enantiomer-specific BABA receptor that interacts with the transcription factors VOZ1 and VOZ2 [17, 18]. In BABA -primed cells, this interaction represses the expression of ABA genes, resulting in increased expression of PTI genes and callose-associated defence [17]. The previous study of our laboratory showed that, as in potato, effective BABA-IR is also associated with the formation of HR -like lesions in tomato [19]. However, while BABA-IR appears to activate SA signalling pathways in *Arabidopsis* and potato plants, our results suggest that ET signalling pathways play a key role in BABA-IR in tomato plants [19].

Here, we present a study in which a combination of nontargeted approaches was used to elucidate the molecular basis of BABA-IR in tomato (*Solanum lycopersicum* cv. *Marmande*), an important crop [20]. BABA was applied by spraying onto leaves, as this application strategy is easy to implement in practical agriculture. Global transcriptomic and proteomic analysis of tomato plants allowed us to identify the molecular processes and signalling pathways that occur in tomato at BABA-IR.

## Results

### Growth of pathogens having different lifestyles after BABA treatment

**Figure 1:**
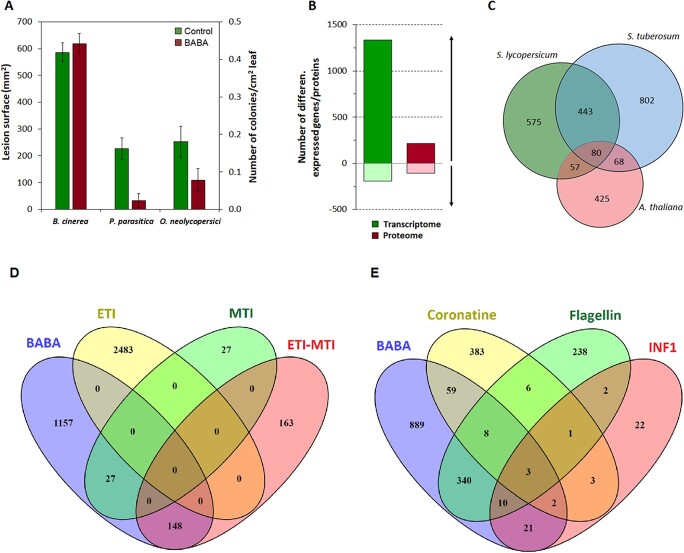
Resistance, Transcriptomic, and Proteomic changes associated with BABA-IR, overlap between BABA-induced genes in different plant species and overlap between genes induced by BABA and selected elicitors. (A) Lesion surface area in 6–8 weeks-old tomato plants after spraying with 10 mM followed by challenge with the phytopathogens *Botrytis cinerea*, *Phythophtora parasitica*, and *O. neolycopersici*. (B) Total numbers of differentially expressed genes and proteins during BABA-IR (calculated as the ratio of the expression in 10 mM BABA-sprayed and water-sprayed leaves) 24 h after treatment. Cut-offs of a ≥ 2-fold difference in expression and P ≤ 0.01 (for genes) or P ≤ 0.05 (for proteins) were applied. The number of genes or proteins in each category is shown. (C) Degrees of overlap between orthologous groups identified in this study and previous transcriptomic studies on BABA-treated potato (Bengtsson et al., 2014) and *Arabidopsis* plants (Zimmerli et al., 2008). (D) Overlap between genes induced in BABA-treated tomato Marmande plants and in tomato Rio Grande (RG)-PtoR resistant plants during MTI, ETI, or both (Pombo et al., 2014). (E) Overlap between genes induced in BABA-treated tomato Marmande plants and in tomato plants treated with the virulence factors coronatine (COR) from *Pst* DC3000 (Geng et al., 2014), flagellin from *P. syringae* (Rosli et al., 2013), and elicitin INF1 secreted by *P. infestans* (Kawamura, 2009). Induced genes were identified by applying cut-offs of a ≥ 2-fold difference in expression and P < 0.05.

To determine the protective effect of BABA treatment towards pathogens with different lifestyles (biotrophic *Oidium neolycopersici*, hemibiotrophic *Phytophthora parasitica*, and necrotrophic *Botrytis cinerea)* in *S. lycopersicum* cv. *Marmande* plants, we treated plants with 10 mM BABA. This dose was selected based on a previous study [21] showing maximal (95%) protection against *Phytophthora infestans* with no effect on the general growth of the *S. lycopersicum* cv. F1 hybrid cv Baby plants. Indeed, BABA was unable to trigger effective resistance, in our experimental conditions, below concentration of 5 mM, as shown previously [21]. More than two days after treatment we observed not reproducibly the appearance of HR-like microlesions on some BABA spayed leaves (Fig S1) not connected to plant age or leaf position, as described previously [21]. BABA treatment significantly reduced the sporulation of *O. neolycopersici* and the spreading of *P. parasitica* but had no effect on *B. cinerea* disease (Fig 1A). These findings are in agreement with previous studies showing that BABA-IR to biotrophic and hemibiotrophic pathogens in tomato and potato plants but gave inconsistent results against the necrotrophic pathogen *B. cinerea*, possibly due to the use of different methods to evaluate resistance [22–24].

### Transcriptome and proteome modifications during BABA-IR in tomato

Three leaf biological replicates were sampled 24 h and 48 h after spraying with 10 mM BABA. Six samples collected at 24 hrs were used for RNA sequencing and six samples collected at 48 hrs were used for label-free LC–MS/ analysis of the proteome excluding microsomal fraction (thereafter proteome). RNA sequencing generated 334 275 668, high-quality reads with an average of 55 712 611 reads per sample (Table S1). The reads for each sample were mapped to the *S. lycopersicum* reference genome sequence, with 74% of reads being mapped successfully. Only genes with a median count above 20% in at least one treatment were considered in the subsequent analysis. The cut-offs used in the comparison were P < 0.01, and ≥ 2-fold expression change. Using these criteria, we identified 24 562 genes from 34 725 annotated genes (ITAG 2.4), with 1523 genes being differentially expressed (Fig 1B, Table S2). In the proteome analysis, protein identification was only performed for peptides of at least six amino acids with a statistically significant peptide score (q < 0.01, FDR 1%; FDR based on decoy search against the reverse database). The cut-offs used for the comparison were q < 0.05 and ≥ 2-fold expression change. Using these criteria, we identified 1808 protein groups (Table S3) and 319 differentially expressed proteins (Fig 1B, Table S2). As with the transcriptome, we found that far more proteins were upregulated (67%) than downregulated (33%) after BABA treatment (Fig 1B, Table S2), however the correlation between proteins and transcripts changes was only about 10% as discussed previously [25]. These results confirm that BABA treatment causes extensive reprogramming of cellular processes in tomato plants, as previously observed in *Arabidopsis* and potato [22, 26] . However, a comparison of orthologous groups between our transcriptomic study and previous studies on *Arabidopsis* [26] and potato [22] plants exposed to BABA revealed significantly greater overlap between the potato and tomato datasets than between *Arabidopsis* and tomato (Fig 1C, Table S4).

### BABA exhibits common features with MAMPs-triggered immunity in tomato

Sets of genes whose transcript abundance was specifically increased at 6 hours post-infection (hpi) during MAMPs-triggered immunity (MTI) and effector-triggered immunity (ETI) were recently identified using RNA-Seq technology in tomato Rio Grande (RG)-PtoR resistant plants [27]. Interestingly, almost 50% of the BABA-upregulated genes were also differentially regulated during MTI, but there was no overlap between ETI-upregulated genes and BABA treatment (Fig 1D, Table S5). Following this result, the response of tomato plants to BABA was far more similar to that induced by exposure to a well-characterized MAMP flagellin flgII-28 from the bacterium *Pseudomonas syringae* [28] or elicitin INF1 secreted by *P. infestans* [29] than to the response induced by the virulence factor coronatine (COR) secreted by *Pst* DC3000 [30] (Fig 1E, Table S5). Specifically, almost 48% and 57% of BABA-upregulated genes were also upregulated following exposure to flgII-28 analysed using RNA-Seq technology at 6 hpi [28] and INF1 analysed using a GeneChip tomato genomic array 12 hpi [31], respectively. Almost 85% of COR-upregulated genes analysed using a TOM1 cDNA microarray 24 hpi of tomato var “Glamour” seedlings [32] were unaffected by BABA treatment (Fig 1E, Table S5). The only genes upregulated by both BABA and COR were associated with ET (ACC synthase and ACC oxidase) and JA signalling pathways.

### BABA functions as a stress factor

A gene ontology (GO) term enrichment analysis was performed to identify critical processes upregulated by BABA treatment. The sets of terms obtained using the transcriptomic and proteomic data were similar (Fig S2, Table S6), with many common terms in the GO categories “Process” and “Function”.

**Figure 2:**
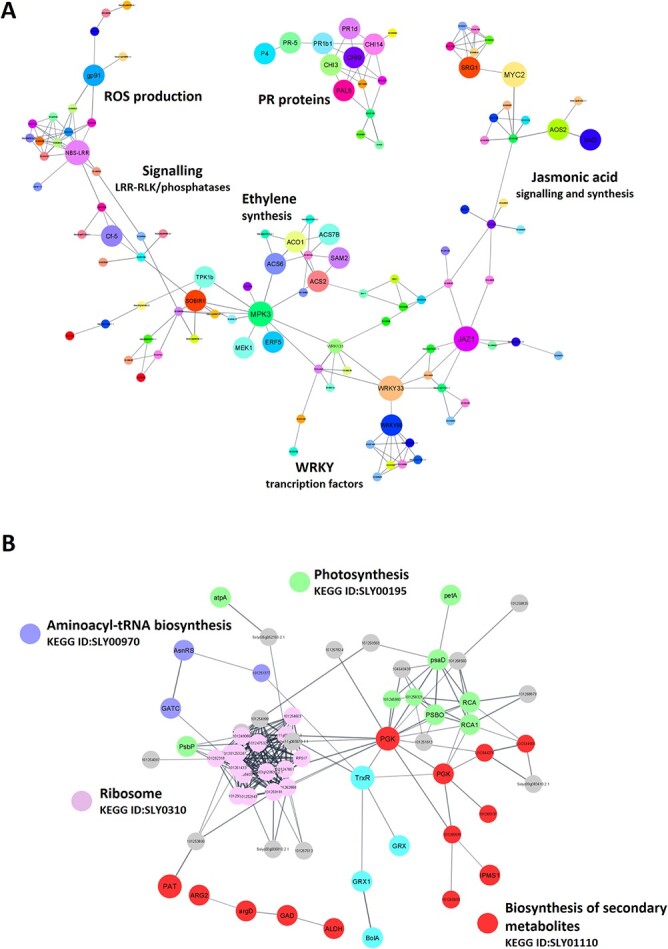
Transcriptomic and proteomic comparisons of plants treated with BABA revealed the existence of sets of genes and proteins that are specifically induced during BABA-IR. Protein–protein association networks were generated for significantly induced (A) genes and (B) proteins, applying cut-offs of a ≥ 2-fold difference in expression and P ≤ 0.01 (for genes) or P ≤ 0.05 (for proteins). Interaction networks were constructed in STRING using a minimum required interaction score of 0.7 [33] and visualized with Cytoscape [94].

The protein–protein interaction network based on RNA-Seq data was generated by directly mapping upregulated genes to proteins in the String database [33] (Fig 2A, Table S7). The network is highly aggregated with clustered sub-networks comprising proteins associated with defence responses (PR proteins), JA and ET signalling and synthesis, regulation of transcription related to mitogen-activated protein kinase MPK3, and processes related to reactive oxygen species (ROS) production. This is also consistent with maps produced after the ReviGO [34] analysis showing significant enrichment of stress-associated clusters (Fig S2, Table S6). The protein–protein interaction network based on proteins exhibiting significant changes in abundance 48 hours after BABA treatment (Fig 2B, Table S7) featured notable clusters relating to photosynthesis, secondary metabolite biosynthesis, and translation. The photosynthesis cluster shows that BABA induces complex changes in the regulation of photosynthesis-related energetic processes and carbohydrate metabolism. The cluster related to secondary metabolites includes the enzymes prephenate aminotransferase (PAT), arginase (ARG2), and lactate dehydrogenase. PAT plays a role in the biosynthesis of aromatic amino acids, while lactate dehydrogenase plays an important role in detoxifying D-lactate, a product of the glyoxalase pathway for detoxifying methylglyoxal, which accumulates under stress conditions [35]. The arginase in tomato leaves was suggested to be involved in ROS homeostasis when its expression was induced by JA signalling following wounding and exposure to *P. syringae* pv. *tomato* strain DC3000 [36]. The upregulation of the proteins in the cluster related to translation is probably related to translational switching from growth to defence [37] and supports the reported role of non-canonical functions of aminoacyl-tRNA synthetase in BABA responses [18].

**Figure 3:**
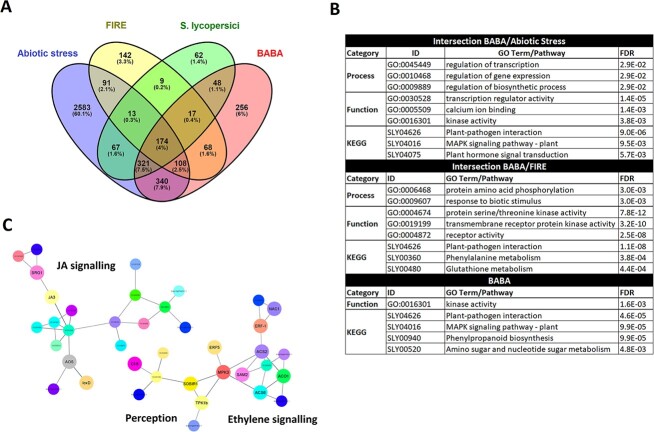
Overlap between genes induced by BABA and genes induced in tomato plants subject to various abiotic and biotic stresses. (A) Overlap between genes induced in BABA-treated tomato Marmande plants and genes upregulated in tomato plants subject to abiotic heat stress [38], genes upregulated in abiotic salinity stress [39], genes upregulated following infection by the fungus *Stemphylium lycopersici* (biotic stress; [40]), and FIRE (flagellin-induced repressed by effectors) genes, representing a pathogen-defined core set of immune-related genes [28]. Cut-offs of a ≥ 2-fold difference in expression and q < 0.05 were applied. (B) KEGG and Gene Ontology (GO) term analysis were performed for genes in each category. The most-enriched GO terms in the process and function categories are shown. (C) Protein–protein association network for genes significantly induced by both BABA treatment and abiotic stress. The interaction network was constructed in STRING using a minimum required interaction score of 0.7 [33] and visualized with Cytoscape [94].

To distinguish between stress-related genes upregulated following BABA treatment and defence-related genes involved in BABA-IR, we compared the BABA-upregulated genes to the sets of genes upregulated in previous transcriptomic studies on tomato plants subjected to temperature [38] and salinity [39] stress as well as those upregulated in tomato plants infected by the fungus *Stemphylium lycopersici* [40] and the FIRE (flagellin-induced repressed by effectors) genes, which represent a pathogen-defined core set of immune-related genes [28]. We found that 50% of the BABA-upregulated genes overlapped only with the sets of genes upregulated by abiotic stress, confirming the hypothesis that BABA acts on tomato plants primarily as a stress factor. However, 30% of the BABA-upregulated genes were also FIRE genes or genes upregulated following *S. lycopersici* infection, suggesting that these genes are associated with BABA-augmented defence expression against *P. parasitica* and *O. neolycopersici*. Moreover, about 20% of genes unique to the BABA treatment were significantly enriched in KEGG pathways related to plant-pathogen interaction, MAPK signalling, and phenylpropanoid synthesis, indicating that these genes are also involved in BABA-IR (Fig 3B, Table S5).

### The BABA stress response is orchestrated via ethylene and jasmonic acid signalling

**Figure 4:**
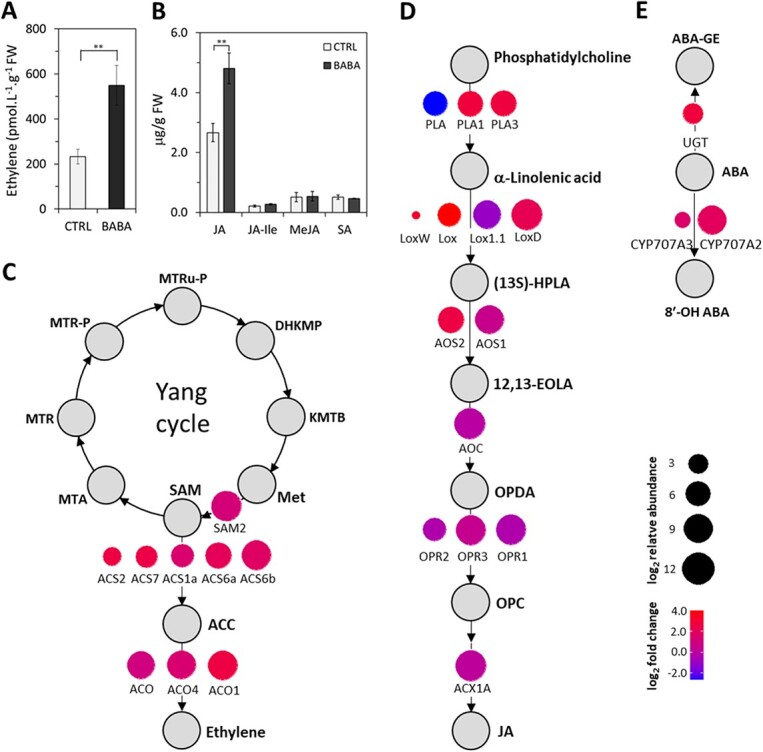
Involvement of signalling pathways in BABA-IR. (A) ET accumulation was measured at 24 hrs after BABA treatment (10 mM) of leaflets (n = 8) by gas chromatography. (B) Levels of JA, jasmonic acid-isoleucine (JA-Ile), methyl-jasmonate (MeJA) and SA were measured by LC–MS 24 h after BABA treatment (10 mM) leaves (n = 6). The control tissue (CTRL) was a water-treated control sample. Each bar represents the mean ± SE. Asterisks denote mean values that differ significantly from that for the control group based on Student’s t-test at P ≤ 0.01 (**). (C-E) The BABA-induced induction of genes involved in the synthesis of ET (C) and JA (D) and the degradation of ABA (E) is shown.SAM – S-Adenosyl-L-methionine, MTA – 5′-Methylthioadenosin, MTR – 5′-Methylthioribose, MTR-P – 5′-Methylthioribose-1-phosphate, MTRu-P – 5′-Methylthioribulose-1-phosphate, DHKMP – 1,2-Dihydroxy-3-keto-5-methylthiopentene, KMTB – 2-keto-4-methyl-thiobutyrate, SAM2 – S-Adenosyl-L-methionine synthase, ACS – 1-aminocyclopropane-1-carboxylic acid synthase, ACC – 1-aminocyclopropane-1-carboxylic acid, ACO – 1-aminocyclopropane-1-carboxylic acid oxidase, PLA – Phospholipase A, Lox – Lipoxygenase, (13S)-HPLA – 13-hydroperoxylinolenic acid, AOS – Allene oxide synthase, 12,13-EOLA – 12,13-epoxyoctadecatrienoic acid, AOC – Allene oxide cyclase, OPDA – (9S,13S)-12-oxo-phytodienoic acid, OPR – 12-oxo-phytodienoic acid 10,11 reductase, OPC – 3-oxo-2-(2′-pentenyl)-cyclopentane-1-octanoic acid, ACX1A – acyl-CoA oxidase, JA – Jasmonic acid, ABA – Abscisic acid, UGT – Abscisic acid glucosyl-transferase, ABA-GE – Abscisic acid glucosyl ester, 8´-OH ABA – 8′-hydroxy abscisic acid N.C. – transcript not changed, N.D. – transcript not detected.

The preceding analysis revealed a clear enrichment of genes associated with JA and ET signalling pathways. Moreover, both ET and JA accumulated in tomato plants during the first few hours after BABA treatment (Fig 4A, B). Accordingly, BABA induced upregulation of several isoforms of ACC synthase (*ACS*) and ACC oxidases (*ACOs*) (Fig 4C, Table S8), essential enzymes for ET biosynthesis [41, 42]. The high upregulation of the *ACS2* and *ACS6* isoforms is also consistent with their reported activation during defence reactions in *Arabidopsis* [41] and with the observed upregulation of *WRKY33*, which activates *ACS2* and *ACS6* expression downstream of the *MPK3/MPK6* cascade [42]. Our data also support the findings of an earlier study on *Arabidopsis* [43] showing that the conversion of methylthioadenosine (MTA) to Met via the Yang (or Met salvage) cycle is generally not controlled by ET signalling because BABA treatment had no detectable effect on the regulation of the Yang cycle genes *MTN*, *MTK*, *MTI*, and *ARD* (Fig 4C, Table S8).

Among the BABA-upregulated genes were the patatin-like proteins PLA1 and PLA3 (Fig 4D, Table S8), which have been implicated in wound responses and resistance to necrotrophic pathogens via JA signalling [44]. In addition, there was significant upregulation of genes encoding enzymes involved in the synthesis of endogenous JA (*TomloxD*, *AOS2* and *OPR3*). The upregulation of these genes was accompanied by the accumulation of JA in BABA-treated leaves (Fig 4D). Also upregulated were 6 of the 12 Jasmonate ZIM Domain (*JAZ*) genes, key regulators of JA signalling that govern host and non-host pathogen-induced cell death in tomato. The most highly upregulated *JAZ* genes (*SlJAZ1*, *SlJAZ2* and *SlJAZ9–11*) were also induced by treatment with COR [45].

Surprisingly, unlike in previous studies on the effects of BABA in *Arabidopsis* [14] and potato plants [22], there was no significant enrichment of genes associated with the phytohormone SA. In keeping with this result, the levels of total SA declined slightly in leaves treated with BABA (Fig 4B). A recent study showed that ABA signalling is suppressed during BABA-IR in *Arabidopsis* plants [17]. In accordance with this result and our previous study [46], we observed no enrichment of genes associated with ABA signalling following BABA treatment in tomato and several genes involved in ABA catabolism were upregulated including *CYP707A* (*Solyc08g005610.2.1*) and UDP-glucosyltransferase (*UGT*; *Solyc09g098080.2.1*) (Fig 4E, Table S8).

### BABA upregulates perception and signalling machinery related to abiotic stress

Protein kinases comprised 142 of the BABA-upregulated genes (Fig S3, Table S9), accounting for 15% of the total set of expressed kinases in our analysis. Similarly, 44 protein kinases were identified in the proteomic analysis, of which 14 were upregulated and only 3 were downregulated (Fig S3). These results suggest that protein kinases play an important role in the BABA stress response (Fig S3, Table S9). An enrichment analysis of the BABA-responsive families using the chi-squared test revealed overrepresentation of the large Receptor-Like Kinase/Pelle (RLK-Pelle) family, which is crucial for plant-specific adaptation [47] (Fig S3) at both the transcriptomic and proteomic levels. This finding agrees well with the upregulation of 27 Receptor-Like Proteins (RLP), representing 23% of the total expressed RLPs in our analysis. It is also notable that all previously reported PRRs in tomato plants were upregulated including those for flagellin (*FLS2* and *FLS3*), the fungal elicitor *EIX* (*LeEix1* and *LeEix2*), *Ave1* from *V. dahlia* (*Ve1* and *Ve2*) [48], and *Avr* factors (*Cf-2*, *Cf-4*, *Cf-5* and *Cf-9*) [49]. This increased expression of perceptual proteins is consistent with the observed protein–protein interaction between *WRKY33* and the *MPK3* kinase from the CMGC family (Fig 2B, Fig S3). The *MPK3/MPK6* cascade causes the phosphorylation of *WRKY33* and the closely related *WRKY25*, leading to ET production due to activation of the enzyme *ACS* as described above. Two other RLKs interacting with RLPs involved in MAMPs perception, *SOBIR1* [50] and *TARK1* [51], were also upregulated.

In parallel with the induction of pathogen perception machinery, we also observed massive upregulation of genes encoding transcription factors (TFs): BABA treatment induced the upregulation of 130 TF genes (Fig S4). The largest numbers of induced genes were found in the ERF, WRKY, MYB and NAC families (Fig S4), which play essential roles in regulating stress responses in plants, mainly through ET and JA signalling pathways. The strong induction of ERFs following BABA treatment was particularly notable: of the 137 identified ERF genes in tomato [52], 113 were expressed, 33 were upregulated, and 5 were downregulated. The BABA response has many features in common with the responses to cold, salt, and mechanical stress observed in previous studies on tomato [38, 53, 54]. However, the BABA-upregulated genes *SlERF*5 (*ERF*5), *SlERF*43 (*RAV2*), *SlERF*55 (*TSRF*1), *SlERF*60 (*Pti*5) or *SlERF*69 (*ERF*1) were previously linked to the activation of defence responses against diverse pathogens (Table S9). Unlike in previous studies on tomato, no WRKY genes were downregulated after BABA treatment; their expression patterns resembled those induced by salt stress, the tomato spotted wilt virus (TSWV), and the fungal elicitor *EIX* [55]. The largest class of plant MYB factors are the 2R proteins, which regulate primary and secondary metabolism, hormone signal transduction, development, and responses to biotic and abiotic stresses [56]. Of the 91 expressed 2RMYB genes, 11 were upregulated following BABA treatment and 6 were downregulated. Their expression profile most closely resembled that seen in tomato plants treated with MeJA [57] or stressed by infection with the bacterial pathogens *Pst* DC3000 or *Pseudomonas putida* (Table S9).

Finally, a comparison of the BABA-upregulated RLPs/WRKY, ERF, and MYB genes to recent RNA-Seq results for tomato plants under biotic [27] and abiotic stress [38] confirmed that the pattern of upregulation induced by BABA is significantly more similar to that for abiotic stress than that for biotic stress (Table S9).

### Amino acid metabolism in BABA-treated plants

**Figure 5:**
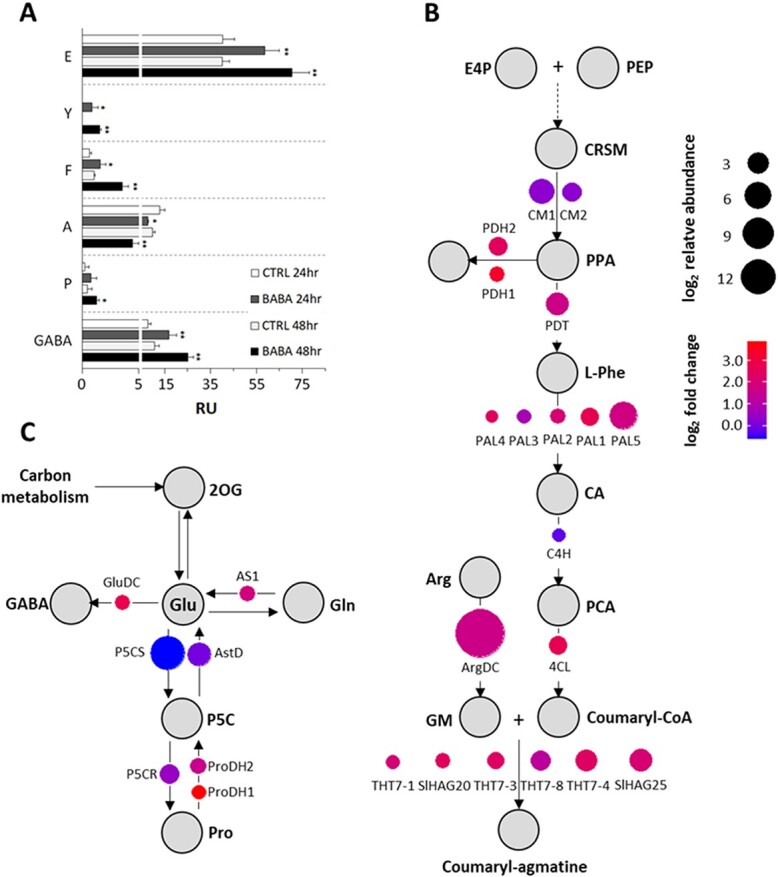
BABA-induced changes in amino acid levels and their metabolic pathways. (A) Amino acids whose abundance in BABA-treated Marmande tomato plants differs significantly from that in water-treated controls. Data are means from three replicates; the errors are standard errors of means. Statistically significant differences recorded for each amino acid as determined by the t-test are indicated with different numbers of asterisks (^*^P < 0.05, ^**^P < 0.01). (B, C) Induction of genes belonging to the phenylpropanoid pathway (B) and glutamate metabolic pathway (C). E4P – erythrose-4-phosphate, PEP – phosphoenolpyruvate, CRSM – chorismic acid, CM – chorismic acid mutase, PPA – prephenate, PDH – prephenate dehydrogenasa, PDT – prephenate dehydratase, PAL – Phenylalanine ammonia-lyase, CA – cinnamic acid, C4H – Cytochrome P450, PCA – p-coumaric acid, 4CL – 4-coumarate CoA ligase, THT – Tyramine N-(hydroxycinnamoyl) transferase, GM – Agmatine, ArgDC – Arginine decarboxylase, 2OG – 2-ketoglutarate, GluDC – Glutamate decarboxylase, GABA – gamma-Aminobutyric acid, AS1 – Asparagine synthase, P5CS – Gamma-glutamyl phosphate reductase, AstD – N-succinylglutamate 5-semialdehyde dehydrogenase, P5C – 1-pyrroline-5-carboxylate, P5CR – Pyrroline-5-carboxylate reductase, ProDH – Proline dehydrogenase, N.C. – transcript not changed, N.D. – transcript not detected.

Transcriptomic and metabolomic studies have consistently shown that amino acid (AA) homeostasis plays a role in stress responses [58, 59]. Moreover, BABA induces the stress-induced morphogenic response (SIMR). In *Arabidopsis*, Asp levels were increased 3-fold by treatment with the active (R)-BABA enantiomer but were unaffected by the (L) enantiomer, suggesting that BABA obstructs canonical AspRS activity [18]. Surprisingly, we observed no change in Asp levels in tomato plants after BABA treatment. However, there was a significant increase in the levels of the enzymes Asparagine-tRNA synthetase (GlnRS) and plastid Glutamyl-tRNA(Gln) amidotransferase (GATC) as well as the Glu, GABA, Pro, Phe, and Tyr, together with a reduction in Ala levels (Fig 2B, 5A). The glutamate family pathway is strongly activated under stress conditions, leading to the accumulation of GABA and proline. GABA is synthesized from Glu by a decarboxylation reaction in response to abiotic stress, viral infection, and herbivore attack [58]; its increased concentration in BABA-treated tomato plants is almost certainly connected to the strong transcriptomic and proteomic upregulation of its key biosynthetic enzyme glutamate decarboxylase (*Solyc04g025530.2.1*) (Fig 5C). Proline, which plays a pivotal role in responses to abiotic stresses, osmotic, salinity, and low temperature stresses, is synthesized predominantly from glutamate by two successive reductions catalysed by P5C synthetase (*P5CS*) and P5C reductase (*P5CR*), with *P5CS* being the rate-limiting enzyme for proline synthesis [60]. However, although BABA treatment increased the abundance of *P5CS*, it did not upregulate *P5CS* transcription. This suggests that the increase is due to a change in post-transcriptional regulation, as observed previously [61]. Conversely, there was significant transcriptional upregulation of proline dehydrogenases (*ProDH*) (*Solyc02g089630.2.1*, *Solyc02g089620.2.1*), which catalyse the catabolic conversion of Pro into the toxic intermediate P5C (Fig 5C, Table S10). Spraying *Arabidopsis* leaves with Pro or P5C causes the formation of HR-like lesions resembling those induced by BABA [62]. We therefore suggest that the lesion formation observed in tomato after BABA foliar spraying [19, 22] spraying can be attributed to the generation of very high local BABA concentrations on leaf surfaces (white deposits) and subsequent P5C accumulation.

BABA treatment also upregulated transcription of branched-chain amino acid aminotransferase (*Solyc03g043880.2.1*) and cysteine desulfurase (*Solyc11g005840.1.1*) (Fig 5C). Both of these enzymes are important in the degradation of branched AAs, whose complete oxidation in the mitochondria allows large amounts of ATP to be generated under stress conditions that impair photosynthesis [58]. BABA treatment also caused upregulation of the glutamine-dependent asparagine synthetase *AS1* (*Solyc06g007180.2.1*) and LHTs, which function as high-affinity transporters of uncharged/acidic AAs in the mesophyll plasma membrane (*Solyc11g066800.1.1*, *Solyc01g111980.2.1*, *Solyc10g055740.1.1*). These changes indicate an effect on overall nitrogen metabolism in the plant (Fig 5C, Table S10). The upregulation of all these proteins was previously observed during chlorosis caused by proteolytic activity and amino acid deamination during *P. syringae* infections [63]. BABA also upregulated four glutamate receptor genes (*SlGLR1.2*, *SlGLR2.1*, *SlGLR2.2* and *SlGLR2.5*) implicated in various processes including the response to aluminium [64] and enhanced drought tolerance in plants [65].

Two prephenate dehydrogenases (*Solyc09g011870.1.1*, *Solyc06g050630.2.1*) and one prephenate dehydratase (*Solyc06g074530.1.1*) were strongly upregulated at the transcript level following BABA treatment, and prephenate aminotransferase was upregulated at the protein level. These changes are clearly connected to the BABA-induced upregulation of the phenylpropanoid pathway and especially its lignin/lignan branch. Important upregulated enzymes of this branch include 4-coumarate CoA ligase (4-CL) and several caffeoyl-CoA O-methyltransferase (COMT), laccases (LAC), and peroxidases (PER), which catalyse the synthesis of several secondary metabolites (Fig 5B). BABA treatment also caused transcript-level upregulation of arginine decarboxylase (*Solyc10g054440.1.1*), which converts arginine into agmatine, a precursor of polyamines including putrescine, spermidine, and spermine (Fig 5B, Table S10). Interestingly, however, we observed no increase in polyamine levels, suggesting that agmatine serves some other metabolic purpose in BABA-treated tomato plants [66]. It could possibly be converted into *p*-coumaroylagmatine; this hypothesis is supported by the upregulation of *4-CL* (*Solyc06g035960.2.1*) and *PAL* together with several hydroxycinnamoyl-CoA:tyramine N-(hydroxycinnamoyl) transferases (*THT1–3*, *THT1–4*, *THT7–1*, *THT7–8*), which are also upregulated during incompatible interactions of *Pst* with tomato [67] (Fig 5B, Table S10).

## Discussion

BABA has long been described as an agent inducing highly effective resistance against a wide spectrum of biotic and abiotic stresses in multiple plant species [9, 14]. Although much has been learned about the perception and molecular action of BABA in *Arabidopsis* [17, 18], many questions remain regarding its mechanisms of action. Since recent studies have shown that during plant immune responses translation is tightly regulated and poorly correlated with transcription [25], as observed here, we characterized BABA-induced changes in both the transcriptome and the intracellular proteome. In our experimental conditions only high BABA concentration after foliar spraying of the crop tomato cultivar Marmande (which would be a viable agricultural application strategy) behaves as a strong stress inducer that does not only triggers highly effective resistance and that deeply remodels transcriptome and subsequent proteome of tomato. Whether BABA primed tomato defences at lower concentrations without any protective effect was not our aim since it cannot be useful for crop protection. Transcriptomic and proteomic analyses showed a similar trend when most of differentially expressed genes and proteins were upregulated and have been linked to general stress responses and defence. Besides, many proteins exhibiting decreased amounts can be linked to the general decrease in the rate of photosynthesis and carbon metabolism under stress conditions as previously described [59]. It is this stress-induced reduction in plant growth induced by BABA that is the major factor limiting its commercial exploitation [18].

Analysis of the genes and proteins upregulated following BABA treatment revealed enrichment of cellular processes related to primary metabolism and responses to stimuli when BABA increased the amount of enzymes involved in carbohydrate metabolism. Previous transcriptomic and genetic analyses have demonstrated the induction of genes involved in carbohydrate metabolism upon challenge by pathogens or PAMPs, and the expression of these genes was shown to affect downstream defence responses including ROS production and PR gene expression [59]. Induction of these genes was also observed in genome-wide studies in *Arabidopsis* plants infected with the avirulent pathogen *P. syringae* pv. Tomato [68] and in rice leaf sheaths infected by *Rhizoctonia solani* [69]. Thus, the genes upregulated in our study and publicly available datasets of potato [22] and *Arabidopsis* [26] (Fig 1C) can be seen as common defence response genes that contribute to BABA-IR.

Previous studies have also shown that BABA-IR is driven by different signalling molecules in different plant species [15, 16, 70, 71]. In *Arabidopsis*, BABA treatment induces accumulation of SA and SA glucoside (SAG) and causes significant changes in the abundance of isochorismate synthase (ICS), which is directly involved in SA biosynthesis which, in turn, is associated with the expression of acidic PR proteins [15, 26]. Although we paid a particular attention to the role of SA in BABA-induced resistance, none of these responses were observed in tomato as in our recent study in which we determined decreased level of SA after BABA treatment of tomato leaflets via petiole aspiration [46]. In addition, unpublished results from our lab, clearly show that SA treatment on tomato leaves failed to induce both a resistance to mildews and the classical defence marker genes outlined in the literature and in this paper (mainly PR-proteins coding genes). It seems that SA is probably not so important in tomato defence to biotic stresses since attempts to protect tomato with SA and SA mimics always failed in available literature with the exception of a protective effect observed towards tomato canker (*Clavibacter michiganensis*) with acibenzolar-S-methyl. Indeed, some plants, like rice or wintergreen, exhibit a high constitutive level of SA or SA methyl ester without being protected from diseases [72] and we have previously demonstrated that SA sensitizes carnation to disease by inhibiting N-benzoate-based phytoalexins biosynthesis [73]. Thus, what was demonstrated in Arabidopsis and even in tobacco should not signify that all the plant kingdom, especially among crops diversity, would follow the same defence regulation scheme. However, treatment of tomato with BABA did affect genes involved in the biosynthesis of JA and ET as well as basic isoforms of PR proteins, which were also observed in an earlier study on BABA-treated potato and tomato plants [22, 46]. This finding is in accordance with impaired BABA-IR towards *P. infestans* in the tomato *def* mutant, which is defective in JA accumulation [71] and pivotal importance of ET in chitosan- and Flg22-induced local and systemic defence responses of tomato plants previously proved in Never-ripe (*Nr*) tomato mutants exhibiting insensitivity to ET in all vegetative tissues due to mutation in SlETR3 receptor [74, 75]. All these findings clearly emphasize the need to study plant biology with a necessary hindsight when comparisons to model plants fail to confirm established mechanisms.

A recent screening of *Arabidopsis* mutations affecting BABA-IR revealed defects associated with the gene *IBI1*, which encodes aspartyl-tRNA synthetase (AspRS). The specific binding of R-BABA to the L-Asp-binding domain of *IBI1* primes the protein for non-canonical defence activity [18]. In accordance with these findings, we found that BABA had no effect on the regulation of the *IBI1* orthologue in tomato plants. However, we found no evidence of any change in aspartate levels driven by the accumulation of uncharged tRNA^Asp^ leading to inhibition of translation activity via phosphorylation of the initiation factor eIF2α [18]. Instead we observed elevated glutamate levels and increased expression of the enzymes GlnRS and GATC, which are involved in tRNA^Gln^ synthesis [76]. Following these findings, BABA treatment of tomato plants upregulated transcription of HSF24 (*Solyc02g090820.2.1*), a HSF-type homologue of TBF1. The heat shock factor(HSF)-like transcription factor TBF1 was proven to play a crucial role in the growth-to-defence switch that activates multiple defence mechanisms and inhibits primary growth and development upon pathogen challenge [77]. Interestingly, one suggested activation mechanism for TBF1 is related to the GCN2-dependent phosphorylation of eIF2α, which is regulated via the accumulation of uncharged tRNAs. This is consistent with the reported inhibitory activity of R-BABA towards the cellular AspRS activity of *IBI1* in *Arabidopsis* [18]. Moreover, TBF1 controls distinct output genes in SAR and MTI, which could be connected to our observation that BABA-IR in tomato is mediated by genes involved in MTI. Together, these observations convincingly explain the finding that BABA has dual activities in tomato, simultaneously activating defence mechanisms and downregulating protein synthesis and carbon metabolism. The latter activity is similar to the stress responses [59] associated with SIMR in *Arabidopsis*. It has been suggested that BABA primes defence responses simply via this stress imprinting process because L-glutamine treatment reduced both BABA-induced SIMR and BABA-IR [78]. In keeping with this hypothesis, 70% of the BABA-induced transcripts overlapped with the set of transcripts upregulated by abiotic salt [39].

Two important processes in pathogen recognition and the subsequent activation of plant defence mechanisms are the secretion and spotting of diverse pattern-recognition receptors (PRRs) to the plasma membrane and the activation of protein kinases involved in signal transduction cascades. Augmented perception of stress signals by plant cells seems to be essential in BABA-IR, as demonstrated by the significant enrichment of GO terms and pathways related to receptor activity after BABA treatment. Moreover, extensive induction of signalling and perception machinery was one of the main factors distinguishing BABA-treatment from the other stress conditions and put forward in our data set. BABA induced a significant number of receptor and receptor-like kinases involved in abiotic stress responses (L-type lectin receptor kinases) [79], MAMPs perception, *Phytophthora* resistance (*LysM*, *Bti9*, *SOBIR1*) [50, 80], and responses to pathogen infection, mechanical wounding, and oxidative stress (*TPK1b*) [81]. This is the first demonstration that BABA-IR in tomato is connected to a hyper-receptive status. In that way, BABA acts as a real priming agent, preparing the plant to rapidly recognize pathogens and to set-up strong defences.

**Figure 6:**
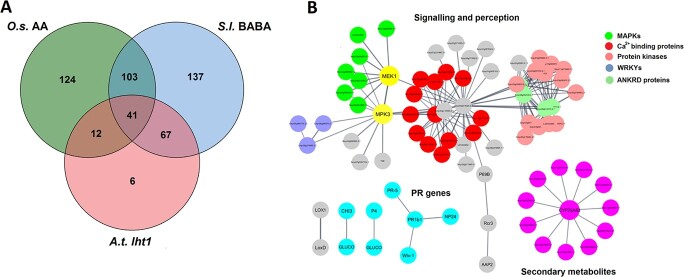
Overlap between genes induced by BABA and genes induced in selected plants with altered amino acid homeostasis. (A) Degrees of overlap between orthologous groups identified in this study and previous transcriptomic studies on *A. thaliana* lht1 plants [82], and Glu-treated rice plants [83]. (B) Functional enrichment of the plant-pathogen interaction pathway (KEGG) and protein domains related to signal transduction (INTERPRO) among the orthologous genes common to all three sets.

However, as stated above, BABA also induce a major plant stress. Whether these two aspects can be disconnected is a pending question to only keep the hyper-receptive side unless this status could be a consequence of the major stress. Plant defence responses are also modulated by AA homeostasis and treatment with high concentrations of AAs. For example, the *A. thaliana lht1* mutant, which has reduced levels of Gln, Ala, and Pro, exhibits SA dependent resistance to a wide range of diseases [82]. In addition, treatment of rice roots with the AAs Glu, Asn, Met, and Asp induced systemic disease resistance against rice blast that was partially dependent on SA signalling and did not cause any change in the content of free AAs in leaves [83]. In our experiments, treatment with BABA increased levels of Pro and the expression of the Glu biosynthetic enzyme ProDH, as well as the levels of free Glu in the leaves (Fig 5A, C). The ProDH is an enzyme that plays a crucial role in plant metabolism. Recent studies have shown that ProDH activity is upregulated in response to pathogen infection and contribute to HR and disease resistance, which apparently potentiates the accumulation of ROS. In addition, ProDH may also regulate the balance between proline and P5C, which has been shown to affect the accumulation of defence-related metabolites and the expression of defence genes [84–86]. Decreases in Glu and Pro levels are also associated with microbial community breakdown and disease incidence, supporting the idea that they play an important role in the plant’s defence response [87]. Free Glu may be recognized by glutamate receptor-like proteins (GLRs), which have been implicated in enhanced resistance to *Hyaloperonospora arabidopsidis*, and *P. syringae* in *Arabidopsis* [88]. The initial cellular events in BABA-IR in tomato may thus involve GLRs as suggested previously in a study on AA-ISR to rice blast in leaves [83]. Interestingly, despite significant differences, the upregulated gene clusters in BABA-treated tomato overlap extensively with the sets of orthologous upregulated genes identified by microarray analysis of *A. thaliana lht1* plants [82] and the genes upregulated in Glu-treated rice plants [83] (Fig 6A). Notably, the orthologous genes common to all three sets exhibited functional enrichment in the plant-pathogen interaction pathway (KEGG) and in protein domains related to signal transduction (INTERPRO), suggesting that, in all three cases, induced resistance is driven by similar molecular mechanisms based on sensitization to stress responsiveness (Fig 6B), which may be characteristic of priming phenomena [3].

Collectively, here we demonstrate that a strong BABA-IR towards *Phytophthora parasitica* and *Oidium neolycopersici* in tomato cv Marmande resemble in many aspects responses to general stress. This resistance was largely explained by the activation of the ET and JA pathways resulting in a strong defence set-up involving PR-proteins as well as phenyl propanoid pathway, lipid peroxidation but in the same time revealed a complete remodelling of plant functions including decrease in primary metabolism and in photosynthesis together with an enhanced ability to perceive (P)(M)(D)AMPs and to set-up downstream signalling. In conclusion, much more attention should be paid to comparative studies between plants of agronomic interest submitted to R-BABA treatment. We clearly evidenced that multiomics as well as targeted approaches can bring original insight on who to who, even though many black boxes still remain closed.

## Materials and methods

### Plant material and growing conditions

Tomato plants (*S. lycopersicum* cv. *Marmande*) were grown at 75% humidity and 14 hours of light (day 24°C, night 22°C). After 6 or 7 weeks of growth, whole tomato plants were sprayed with 10 mM DL-BABA or water. Leaflets were then removed from plants 24 or 48 hours after spraying for transcriptome and proteome analysis and processed immediately or stored at −80°C until use. Plants assigned to each treatment were randomly selected, labelled, and then returned to the growth chamber. Three biological replicates were selected for each treatment.

### *Botrytis cinerea* and *Phytophthora parasitica* inoculation and measurement

Two days after spraying with 10 mM BABA or water, leaflets were removed from 7–8 week-old tomato plants and placed in clear Styrofoam boxes with moist absorbent paper to maintain high relative humidity. The centre of each leaflet was inoculated with a mycelial plug (5 mm in diameter) from the growth margin of a 3-day-old culture of the BC21 strain of *B. cinerea*. Alternatively, leaflets were pricked with a needle at a marked spot and 20 μl of a *P. parasitica* zoospore suspension (40 000 zoospores/ml) was applied to the wounded spot. Five replicates in styrofoam boxes with 3 leaflets each were used. After inoculation, the detached leaflets were incubated in a growth chamber under conditions conducive to disease development (21°C, 14-h photoperiod, 114 μmol.s-1.m-2). Symptoms were recorded after 3 days of incubation for *P. parasitica* or 4 days for *B. cinerea*. Photographs were analysed using the ImageJ image analysis programme to quantify the surface area of necrotic lesions (in mm^2^). Analysis of variance was used to evaluate whether differences between controls and treatments were significant for each of the three independent experiments.

### *Oidium neolycopersici* inoculation and measurement

Spraying with 10 mM BABA or water was done on whole 5–6 week old tomato plants. Two days later, each plant was inoculated with approximately 10 ml of a spore suspension of *O. neolycopersici* at a concentration of 10^4^ sp/ml. The inoculated plants were then incubated in a growth chamber under conditions conducive to disease development (21°C, RH > 80%, 14 hours of light). The number of powdery mildew colonies was counted 14 days after inoculation on 2 leaves per plant with 5 plants per test. Three independent tests were performed.

### Identification and quantification of proteins by LC–MS/MS

Three biological replicates in the form of separate plants were subjected to analysis. For each replicate, 4 g of leaflets from 3 different plants were harvested on ice and homogenised at 4°C in 20 ml of extraction buffer (50 mM Tris-Mes (pH = 8.0), 20 mM EDTA, 500 mM sucrose, 10 mM DTT, 100 mM PMSF, cOmplete Mini Protease Inhibitor Cocktail tablets) using an Ultraturrax homogenizer (IKA, DE) at 15000 rpm. Samples were filtered through Miracloth and centrifuged at 20000 x g and 4°C. The supernatant was collected and centrifuged in a Beckman Optima (Beckman-Coulter) ultracentrifuge at 35000 rpm and 4°C with a Ti45 rotor. The supernatant was collected and concentrated using Vivaspin® 3 kDa (GE Healthcare) sample concentrators. The concentrated samples were dialyzed overnight to 10 mM ammonium acetate and finally concentrated to 0.5 ml using 4 ml Amicon® Ultra 4 3 kDa (Merck Millipore Ltd.) sample concentrators. Each sample was then fractionated into 5 fractions by HPLC using an IEX PolyWAX LP mixed bed column (200 x 4.6 mm, 5 μm particles, PolyLC Inc., Columbia, USA) and a gradient of ammonium acetate. The collected fractions were dried under vacuum and subjected to LC–MS /MS analysis (Table S3). The dried protein fractions were processed using a philtre filter-aided sample preparation (FASP) method [81]. LC–MS /MS analyses of the peptide mixtures were performed using the RSLCnano system connected to the Orbitrap Elite hybrid spectrometer (Thermo Fisher Scientific). For more details, see the supplemental material (Appendix S1).

### RNA sequencing analysis

Total RNA was isolated using TRIzol reagent (Life Technologies, Grand Island, NY, USA) and checked for integrity on a Bioanalyzer 2200 (RIN ≥ 7.40). Libraries for sequencing were prepared according to a standard protocol for the SOLiD 5500 system (Life Technology). Sequencing was performed using the SOLiD 5500 W platform. Raw reads of 75 bp in length were mapped to the *S. lycopersicum* build 2.40 reference using ITAG2.4 as the gene model in colour space with the Maxmapper algorithm implemented in Lifescope software (Life Technologies, Ltd). RNA content was assessed using a whole-transcriptome workflow with the quality threshold set to 10, resulting in an assignment probability of greater than 90. The raw sequencing data with corresponding metadata are available in the NCBI Gene Expression Omnibus (GEO) repository under accession number GSE108421. Analytical comparison between BABA and the control treatments was performed using the DESeq package [89].

### Orthology and gene ontology enrichment analysis

Orthologous gene clusters were compared and annotated using the OrthoVenn web platform [90], and the results obtained were visualised using the eulerr R package [91]. Significantly differentially expressed genes and proteins were analysed by Singular Enrichment Analysis (SEA) for GO term enrichment using agriGO [92] based on GO terms retrieved from the PLAZA 3.0 database [93]. Summarisation and visualization of SEA were done using REViGO [34] and Cytoscape [94] with DyNet [95]. Protein–protein association networks for significantly differentially expressed genes and proteins were generated using the STRING database with an interaction score of 0.9 [33] and visualized using Cytoscape [94].

### HPLC analysis of amino acids

Twenty-four hours after treatment with BABA, tomato leaves were collected, frozen in liquid nitrogen, and ground to a fine powder. A portion of 250 mg of this powder was then extracted with 1 ml of extraction buffer (0.1 M HCl and 4.6 μg/ml L-2-aminoadipic acid as an internal standard), mixed thoroughly, incubated on ice for 5 minutes, and centrifuged. A 500 μl aliquot of the resulting supernatant was then diluted with 100 μl methanol and loaded onto an SPE C18 column to adsorb interfering secondary metabolites, which had previously been wetted with 1 ml MeOH and equilibrated with 20% MeOH in 0.1 M HCl. The sample was loaded and the column was washed with 400 μl of 20% MeOH in 0.1 M HCl. Amino acids were recovered in both the flow-through and wash fractions and derivatized and analyzed as previously described [96]. See supplemental material (Appendix S1) for further details.

### Quantitative analysis of salicylic acid, jasmonic acid, and jasmonic acid-isoleucine

The tomato leaflest (100 mg) was frozen immediately after the harvest using liquid nitrogen, and the frozen materials ground under liquid nitrogen and extracted with 750 μl of MeOH-H_2_O-HOAc (90:9:1, v/v/v) containing 100 ng of o-anisic acid as an internal standard. The mixture was centrifuged at 10000 x g for 1 min, the supernatant was collected, and the pellet was repeatadly extracted. The pooled supernatants were dried under nitrogen, resuspended in 200 μl of 0.1% HOAc in H_2_O-MeOH (90:10, v/v), and a portion of the mixture (2–5 μl) was subjected to LC–MS analyzes using a TOF mass spectrometer (Agilent Technologies) as previously described [97]. Further details can be found in the supplemental material (Appendix S1).

### Measurement of ethylene production

Single tomato leaflets 24 hours after treatment with BABA or water were placed in 20 ml test tube when the cut end of the petiole was in sterile water and sealed with a air-tight rubber syringe cap. ETwas accumulated for 4 hours before a 1-ml sample was withdrawn for analysis. ET production was measured using gas chromatography with a flame ionization detector quantified by using a gas chromatograph flame ionization detector (Agilent GC 6890, Agilent Technologies) as previously described [19].

## Acknowledgements

D.P. acknovledges support of the European Regional Development Fund-Project “Centre for Experimental Plant Biology”: No. CZ.02.1.01/0.0/0.0/16_019/0000738 funded by MEYS CR and by the Institutional Research Fund of Masaryk university, MUNI/A/1492/2021. CIISB research infrastructure project LM2018127 funded by MEYS CR are gratefully acknowledged for the financial support of the measurements at the CEITEC Proteomics Core Facility. This work was also supported by INRAE, Département Santé des Plantes et Environnement through the funding of the ELICITOM project. This project was supported by the National Infrastructure France Génomique (Commissariat aux Grands Investissements, ANR-10-INBS-09-03, ANR-10-INBS-09-02).

## Author Contributions

Conceptualization: JL, MP, CR and ZZ; Data curation: MZ, CR, BI, MB, KLB, PN, VM, AS, PB, and DP; Data analysis: MZ, CR, DP, AS and BI; Funding acquisition: JL, MP and ZZ; Methodology: MZ, CR, JL, MP and ZZ; Project administration: JL and MP; Resources: JL, MP and ZZ; Validation: MZ, CR, JL, MP and ZZ; Writing, review, and editing: MZ, JL, and MP; Supervision: JL and MP.

## Data availability

Raw sequencing data with appropriate metadata are available in the NCBI Gene Expression Omnibus (GEO) repository under accession number GSE108421.

The mass spectrometry proteomics data have been deposited to the ProteomeXchange Consortium via the PRIDE partner repository with the dataset identifier PXD038074.

## Conflict of interests

None declared.

## Supplementary Data

Supplementary data is available at *Horticulture Research* online.

## Supplementary Material

Web_Material_uhad068

## References

[1] Savary S , Ficke A, Aubertot J-N et al. Crop losses due to diseases and their implications for global food production losses and food security. Food Secur. 2012;4:519–37.

[2] Jones JDG , Dangl JL. The plant immune system. Nature. 2006;444:323–9. 17108957 10.1038/nature05286

[3] Conrath U , Beckers GJM, Langenbach CJG et al. Priming for enhanced defense. Annu Rev Phytopathol. 2015;53:97–119. 26070330 10.1146/annurev-phyto-080614-120132

[4] Westman SM , Kloth KJ, Hanson J et al. Defence priming in Arabidopsis – a meta-analysis. Sci Rep. 2019;9:13309. 31527672 10.1038/s41598-019-49811-9 PMC6746867

[5] Vijayakumari K , Jisha KC, Puthur JT. GABA/BABA priming: a means for enhancing abiotic stress tolerance potential of plants with less energy investments on defence cache. Acta Physiol Plant. 2016;38:1-14.

[6] Baccelli I , Mauch-Mani B. Beta-aminobutyric acid priming of plant defense: the role of ABA and other hormones. Plant Mol Biol. 2016;91:703–11. 26584561 10.1007/s11103-015-0406-y

[7] Pieterse CMJ , Zamioudis C, Berendsen RL et al. Induced systemic resistance by beneficial microbes. Annu Rev Phytopathol. 2014;52:347–75. 24906124 10.1146/annurev-phyto-082712-102340

[8] Chassot C , Buchala A, Schoonbeek H-J et al. Wounding of Arabidopsis leaves causes a powerful but transient protection against botrytis infection. Plant J Cell Mol Biol. 2008;55:555–67. 10.1111/j.1365-313X.2008.03540.x 18452590

[9] Cohen Y , Vaknin M, Mauch-Mani B. BABA-induced resistance: milestones along a 55-year journey. Phytoparasitica. 2016;44:513–38.

[10] Kuźnicki D , Meller B, Arasimowicz-Jelonek M et al. BABA-induced DNA Methylome adjustment to intergenerational Defense priming in potato to Phytophthora infestans. Front Plant Sci. 2019;10:650, 1-16. 31214209 10.3389/fpls.2019.00650 PMC6554679

[11] Luna E , López A, Kooiman J et al. Role of NPR1 and KYP in long-lasting induced resistance by β-aminobutyric acid. Front Plant Sci. 2014;5:184. 24847342 10.3389/fpls.2014.00184 PMC4021125

[12] Thevenet D , Pastor V, Baccelli I et al. The priming molecule β-aminobutyric acid is naturally present in plants and is induced by stress. New Phytol. 2017;213:552–9. 27782340 10.1111/nph.14298

[13] Baccelli I , Glauser G, Mauch-Mani B. The accumulation of β-aminobutyric acid is controlled by the plant’s immune system. Planta. 2017;246:791–6. 28762076 10.1007/s00425-017-2751-3

[14] Ton J , Jakab G, Toquin V et al. Dissecting the beta-aminobutyric acid-induced priming phenomenon in Arabidopsis. Plant Cell. 2005;17:987–99. 15722464 10.1105/tpc.104.029728 PMC1069713

[15] Zimmerli L , Jakab G, Metraux JP et al. Potentiation of pathogen-specific defense mechanisms in Arabidopsis by beta -aminobutyric acid. Proc Natl Acad Sci U S A. 2000;97:12920–5. 11058166 10.1073/pnas.230416897 PMC18865

[16] Hamiduzzaman MM , Jakab G, Barnavon L et al. β-Aminobutyric acid-induced resistance against downy mildew in grapevine acts through the potentiation of callose formation and jasmonic acid Signaling. Mol Plant-Microbe Interact. 2005;18:819–29. 16134894 10.1094/MPMI-18-0819

[17] Schwarzenbacher RE , Wardell G, Stassen J et al. The IBI1 receptor of β-aminobutyric acid interacts with VOZ transcription factors to regulate Abscisic acid Signaling and callose-associated Defense. Mol Plant. 2020;13:1455–69. 32717347 10.1016/j.molp.2020.07.010 PMC7550849

[18] Luna E , van Hulten M, Zhang Y et al. Plant perception of β-aminobutyric acid is mediated by an aspartyl-tRNA synthetase. Nat Chem Biol. 2014;10:450–6. 24776930 10.1038/nchembio.1520 PMC4028204

[19] Satková P , Starý T, Plešková V et al. Diverse responses of wild and cultivated tomato to BABA, oligandrin and Oidium neolycopersici infection. Ann Bot. 2016;119:mcw188–840. 10.1093/aob/mcw188 PMC5378190 27660055

[20] Home | Food and Agriculture Organization of the United Nations . [cited 15 Feb 2021]. Available: http://www.fao.org/home/en/

[21] Cohen Y . Local and systemic control of *Phytophthora infestans* in tomato plants by dl-3-amino-n-butanoic acids. Phytopathology. 1994;84:55-59.

[22] Bengtsson T , Weighill D, Proux-Wéra E et al. Proteomics and transcriptomics of the BABA-induced resistance response in potato using a novel functional annotation approach. BMC Genomics. 2014;15:315 24773703 10.1186/1471-2164-15-315 PMC4234511

[23] Luna E , Beardon E, Ravnskov S et al. Optimizing chemically induced resistance in tomato against Botrytis cinerea. Plant Dis. 2016;100:704–10. 30688613 10.1094/PDIS-03-15-0347-RE

[24] Worrall D , Holroyd GH, Moore JP et al. Treating seeds with activators of plant defence generates long-lasting priming of resistance to pests and pathogens. New Phytol. 2012;193:770–8. 22142268 10.1111/j.1469-8137.2011.03987.x

[25] Xu G , Greene GH, Yoo H et al. Global translational reprogramming is a fundamental layer of immune regulation in plants. Nature. 2017;545:487–90. 28514447 10.1038/nature22371 PMC5485861

[26] Zimmerli L , Hou B-H, Tsai C-H et al. The xenobiotic beta-aminobutyric acid enhances Arabidopsis thermotolerance. Plant J. 2008;53:144–56. 18047473 10.1111/j.1365-313X.2007.03343.x

[27] Pombo MA , Zheng Y, Fernandez-Pozo N et al. Transcriptomic analysis reveals tomato genes whose expression is induced specifically during effector-triggered immunity and identifies the Epk1 protein kinase which is required for the host response to three bacterial effector proteins. Genome Biol. 2014;15:492. 25323444 10.1186/s13059-014-0492-1 PMC4223163

[28] Rosli HG , Zheng Y, Pombo MA et al. Transcriptomics-based screen for genes induced by flagellin and repressed by pathogen effectors identifies a cell wall-associated kinase involved in plant immunity. Genome Biol. 2013;14:R139. 24359686 10.1186/gb-2013-14-12-r139 PMC4053735

[29] Solanský M , Mikulášek K, Zapletalová M et al. Elicitins’ oligomeric states affect the hypersensitive response and resistance in tobacco. J Exp Bot. 2021;72:3219–34. 33475728 10.1093/jxb/erab011

[30] Geng X , Jin L, Shimada M et al. The phytotoxin coronatine is a multifunctional component of the virulence armament of pseudomonas syringae. Planta. 2014;240:1149–65. 25156488 10.1007/s00425-014-2151-x PMC4228168

[31] Kawamura SHY , Hase S, Takenaka S et al. INF1 Elicitin activates jasmonic acid- and ethylene-mediated signalling pathways and induces resistance to bacterial wilt disease in tomato. J Phytopathol. 2009;157:287–97.

[32] Uppalapati SR , Ishiga Y, Wangdi T et al. The phytotoxin coronatine contributes to pathogen fitness and is required for suppression of salicylic acid accumulation in tomato inoculated with pseudomonas syringae pv. Tomato DC3000. MPMI. 2007;20:955–65. 17722699 10.1094/MPMI-20-8-0955

[33] Szklarczyk D , Morris JH, Cook H et al. The STRING database in 2017: quality-controlled protein-protein association networks, made broadly accessible. Nucleic Acids Res. 2017;45:D362–8. 27924014 10.1093/nar/gkw937 PMC5210637

[34] Supek F , Bošnjak M, Škunca N et al. REVIGO summarizes and visualizes long lists of gene ontology terms. PLoS One. 2011;6:e21800, 1-9. 21789182 10.1371/journal.pone.0021800 PMC3138752

[35] Jain M , Aggarwal S, Nagar P et al. A D-lactate dehydrogenase from rice is involved in conferring tolerance to multiple abiotic stresses by maintaining cellular homeostasis. Sci Rep. 2020;10:12835, 1-17. 32732944 10.1038/s41598-020-69742-0 PMC7393112

[36] Chen H , McCaig BC, Melotto M et al. Regulation of plant Arginase by wounding, jasmonate, and the Phytotoxin Coronatine ^*^. J Biol Chem. 2004;279:45998–6007. 15322128 10.1074/jbc.M407151200

[37] Meteignier L-V , El Oirdi M, Cohen M et al. Translatome analysis of an NB-LRR immune response identifies important contributors to plant immunity in Arabidopsis. J Exp Bot. 2017;68:2333–44. 28369573 10.1093/jxb/erx078

[38] Chen H , Chen X, Chen D et al. A comparison of the low temperature transcriptomes of two tomato genotypes that differ in freezing tolerance: Solanum lycopersicum and Solanum habrochaites. BMC Plant Biol. 2015;15:132, 1-16. 26048292 10.1186/s12870-015-0521-6 PMC4458020

[39] Sun W , Xu X, Zhu H et al. Comparative Transcriptomic profiling of a salt-tolerant wild tomato species and a salt-sensitive tomato cultivar. Plant Cell Physiol. 2010;51:997–1006. 20410049 10.1093/pcp/pcq056

[40] Yang H , Zhao T, Jiang J et al. Transcriptome analysis of the Sm-mediated hypersensitive response to Stemphylium lycopersici in tomato. Front Plant Sci. 2017;8:1257, 1-14. 28769960 10.3389/fpls.2017.01257 PMC5515834

[41] Li G , Meng X, Wang R et al. Dual-level regulation of ACC synthase activity by MPK3/MPK6 cascade and its downstream WRKY transcription factor during ethylene induction in Arabidopsis. PLoS Genet. 2012;8:e1002767, 1-14. 22761583 10.1371/journal.pgen.1002767 PMC3386168

[42] Skottke KR , Yoon GM, Kieber JJ et al. Protein phosphatase 2A controls ethylene biosynthesis by differentially regulating the turnover of ACC synthase isoforms. PLoS Genet. 2011;7:e1001370, 1-13. 21533019 10.1371/journal.pgen.1001370 PMC3080859

[43] Bürstenbinder K , Rzewuski G, Wirtz M et al. The role of methionine recycling for ethylene synthesis in Arabidopsis. Plant J. 2007;49:238–49. 17144895 10.1111/j.1365-313X.2006.02942.x

[44] Canonne J , Froidure-Nicolas S, Rivas S. Phospholipases in action during plant defense signaling. Plant Signal Behav. 2011;6:13–8. 21248491 10.4161/psb.6.1.14037 PMC3121997

[45] Ishiga Y , Ishiga T, Uppalapati SR et al. Jasmonate ZIM-domain (JAZ) protein regulates host and nonhost pathogen-induced cell death in tomato and Nicotiana benthamiana. PLoS One. 2013;8:e75728, 1-7. 24086622 10.1371/journal.pone.0075728 PMC3785428

[46] Janotík A , Dadáková K, Lochman J et al. L-aspartate and L-glutamine inhibit Beta-aminobutyric acid-induced resistance in tomatoes. Plants. 2022;11:2908, 1-12. 36365361 10.3390/plants11212908 PMC9655027

[47] Lehti-Shiu MD , Zou C, Hanada K et al. Evolutionary history and stress regulation of plant receptor-like kinase/Pelle genes. Plant Physiol. 2009;150:12–26. 19321712 10.1104/pp.108.134353 PMC2675737

[48] de Jonge R , van Esse HP, Maruthachalam K et al. Tomato immune receptor Ve1 recognizes effector of multiple fungal pathogens uncovered by genome and RNA sequencing. Proc Natl Acad Sci U S A. 2012;109:5110–5. 22416119 10.1073/pnas.1119623109 PMC3323992

[49] Zhou J-M , Tang D, Wang G. Receptor kinases in plant pathogen interactions: more than pattern recognition. Plant cell. Online. 2017;29:618–37. 10.1105/tpc.16.00891 PMC5435430 28302675

[50] Peng K-C , Wang C-W, Wu C-H et al. Tomato SOBIR1/EVR homologs are involved in Elicitin perception and plant Defense against the Oomycete pathogen Phytophthora parasitica. Mol Plant-Microbe Interact. 2015;28:913–26. 25710821 10.1094/MPMI-12-14-0405-R

[51] Kim J-G , Li X, Roden JA et al. Xanthomonas T3S effector XopN suppresses PAMP-triggered immunity and interacts with a tomato atypical receptor-like kinase and TFT1. Plant Cell. 2009;21:1305–23. 19366901 10.1105/tpc.108.063123 PMC2685636

[52] iTAK. iTAK - Plant Transcription factor & Protein Kinase Identifier and Classifier . [cited 26 Jul 2017]. Available: http://bioinfo.bti.cornell.edu/cgi-bin/itak/index.cgi

[53] Pan Y , Seymour GB, Lu C et al. An ethylene response factor (ERF5) promoting adaptation to drought and salt tolerance in tomato. Plant Cell Rep. 2012;31:349–60. 22038370 10.1007/s00299-011-1170-3

[54] Sharma MK , Kumar R, Solanke AU et al. Identification, phylogeny, and transcript profiling of ERF family genes during development and abiotic stress treatments in tomato. Mol Gen Genomics. 2010;284:455–75. 10.1007/s00438-010-0580-1 20922546

[55] Huang S , Gao Y, Liu J et al. Genome-wide analysis of WRKY transcription factors in Solanum lycopersicum. Mol Gen Genet. 2012;287:495–513. 10.1007/s00438-012-0696-6 22570076

[56] Du H , Wang Y-B, Xie Y et al. Genome-wide identification and evolutionary and expression analyses of MYB-related genes in land plants. DNA Res Int J Rapid Publ Rep Genes Genomes. 2013;20:437–48. 10.1093/dnares/dst021 PMC3789555 23690543

[57] Li Z , Peng R, Tian Y et al. Genome-wide identification and analysis of the MYB transcription factor superfamily in Solanum lycopersicum. Plant Cell Physiol. 2016;57:1657–77. 27279646 10.1093/pcp/pcw091

[58] Hildebrandt TM , Nunes Nesi A, Araújo WL et al. Amino acid catabolism in plants. Mol Plant. 2015;8:1563–79. 26384576 10.1016/j.molp.2015.09.005

[59] Rojas CM , Senthil-Kumar M, Tzin V et al. Regulation of primary plant metabolism during plant-pathogen interactions and its contribution to plant defense. Front Plant Sci. 2014;5:17, 1-12. 24575102 10.3389/fpls.2014.00017 PMC3919437

[60] Hu CA , Delauney AJ, Verma DP. A bifunctional enzyme (delta 1-pyrroline-5-carboxylate synthetase) catalyzes the first two steps in proline biosynthesis in plants. Proc Natl Acad Sci. 1992;89:9354–8. 1384052 10.1073/pnas.89.19.9354 PMC50125

[61] Hua XJ , Van De Cotte B, Van Montagu M et al. The 5′ untranslated region of the at-P5R gene is involved in both transcriptional and post-transcriptional regulation. Plant J. 2001;26:157–69. 11389757 10.1046/j.1365-313x.2001.01020.x

[62] Deuschle K , Funck D, Forlani G et al. The role of Δ1-Pyrroline-5-carboxylate dehydrogenase in proline degradation. Plant Cell. 2004;16:3413–25. 15548746 10.1105/tpc.104.023622 PMC535882

[63] Yang H , Ludewig U. Lysine catabolism, amino acid transport, and systemic acquired resistance. Plant Signal Behav. 2014;9:e28933, 1-4. 25763483 10.4161/psb.28933 PMC4091326

[64] Sivaguru M , Pike S, Gassmann W et al. Aluminum rapidly depolymerizes cortical microtubules and depolarizes the plasma membrane: evidence that these responses are mediated by a glutamate receptor. Plant Cell Physiol. 2003;44:667–75. 12881494 10.1093/pcp/pcg094

[65] Lu G , Wang X, Liu J et al. Application of T-DNA activation tagging to identify glutamate receptor-like genes that enhance drought tolerance in plants. Plant Cell Rep. 2014;33:617–31. 24682459 10.1007/s00299-014-1586-7

[66] Bird CR , Smith TA. The biosynthesis of coumarylagmatine in barley seedlings. Phytochemistry. 1981;20:2345–6.

[67] von Roepenack Lahaye E , Newman M-A, Schornack S et al. P-Coumaroylnoradrenaline, a novel plant metabolite implicated in tomato Defense against pathogens. J Biol Chem. 2003;278:43373–83. 12900412 10.1074/jbc.M305084200

[68] Scheideler M , Schlaich NL, Fellenberg K et al. Monitoring the switch from housekeeping to pathogen defense metabolism in Arabidopsis thaliana using cDNA arrays. J Biol Chem. 2002;277:10555–61. 11748215 10.1074/jbc.M104863200

[69] Mutuku JM , Nose A. Changes in the contents of metabolites and enzyme activities in Rice plants responding to Rhizoctonia solani Kuhn infection: activation of glycolysis and connection to Phenylpropanoid pathway. Plant Cell Physiol. 2012;53:1017–32. 22492233 10.1093/pcp/pcs047

[70] Ton J , Mauch-Mani B. Beta-amino-butyric acid-induced resistance against necrotrophic pathogens is based on ABA-dependent priming for callose. Plant J. 2004;38:119–30. 15053765 10.1111/j.1365-313X.2004.02028.x

[71] Yan Z , Reddy MS, Ryu C-M et al. Induced systemic protection against tomato late blight elicited by plant growth-promoting rhizobacteria. Phytopathology. 2002;92:1329–33. 18943888 10.1094/PHYTO.2002.92.12.1329

[72] Klessig DF , Tian M, Choi HW. Multiple targets of salicylic acid and its derivatives in plants and animals. Front Immunol 2016;7, 1-10. Available: 10.3389/fimmu.2016.00206 PMC4880560 27303403

[73] Ponchet M , Duprez V, Ricci P. SUPPRESSION OF BOTH INDUCED RESISTANCE AND PHYTOALEXIN PRODUCTION BY SALICYLIC ACID DURING ELICITATION OF CARNATION CUTTINGS. In A. M. Kofranek (Eds.), Acta Horticulturae. International Society for Horticultural Science (ISHS): Leuven, Belgium, 1983,61–70.

[74] Czékus Z , Iqbal N, Pollák B et al. Role of ethylene and light in chitosan-induced local and systemic defence responses of tomato plants. J Plant Physiol. 2021;263:153461, 1-12. 34217837 10.1016/j.jplph.2021.153461

[75] Czékus Z , Kukri A, Hamow KÁ et al. Activation of local and systemic defence responses by Flg22 is dependent on daytime and ethylene in intact tomato plants. Int J Mol Sci. 2021;22:8354, 1-21. 34361121 10.3390/ijms22158354 PMC8348740

[76] Pujol C , Bailly M, Kern D et al. Dual-targeted tRNA-dependent amidotransferase ensures both mitochondrial and chloroplastic gln-tRNAGln synthesis in plants. Proc Natl Acad Sci U S A. 2008;105:6481–5. 18441100 10.1073/pnas.0712299105 PMC2359783

[77] Pajerowska-Mukhtar KM , Wang W, Tada Y et al. The HSF-like transcription factor TBF1 is a major molecular switch for plant growth-to-defense transition. Curr Biol. 2012;22:103–12. 22244999 10.1016/j.cub.2011.12.015 PMC3298764

[78] Wu C-C , Singh P, Chen M-C et al. L-glutamine inhibits beta-aminobutyric acid-induced stress resistance and priming in Arabidopsis. J Exp Bot. 2010;61:995–1002. 20007686 10.1093/jxb/erp363 PMC2826644

[79] Wang Y , Weide R, Govers F et al. L-type lectin receptor kinases in *Nicotiana benthamiana* and tomato and their role in *Phytophthora* resistance. J Exp Bot. 2015;66:6731–43. 26248665 10.1093/jxb/erv379 PMC4623685

[80] Miya A , Albert P, Shinya T et al. CERK1, a LysM receptor kinase, is essential for chitin elicitor signaling in Arabidopsis. Proc Natl Acad Sci U S A. 2007;104:19613–8. 18042724 10.1073/pnas.0705147104 PMC2148337

[81] AbuQamar S , Chai M-F, Luo H et al. Tomato protein kinase 1b mediates Signaling of plant responses to Necrotrophic fungi and insect Herbivory. Plant Cell. 2008;20:1964–83. 18599583 10.1105/tpc.108.059477 PMC2518242

[82] Liu G , Ji Y, Bhuiyan NH et al. Amino acid homeostasis modulates salicylic acid-associated redox status and defense responses in Arabidopsis. Plant Cell. 2010;22:3845–63. 21097712 10.1105/tpc.110.079392 PMC3015111

[83] Kadotani N , Akagi A, Takatsuji H et al. Exogenous proteinogenic amino acids induce systemic resistance in rice. BMC Plant Biol. 2016;16:60, 1-10. 26940322 10.1186/s12870-016-0748-x PMC4778346

[84] Fabro G , Kovács I, Pavet V et al. Proline accumulation and AtP5CS2 gene activation are induced by plant-pathogen incompatible interactions in Arabidopsis. MPMI. 2004;17:343–50. 15077666 10.1094/MPMI.2004.17.4.343

[85] Cecchini NM , Monteoliva MI, Alvarez ME. Proline dehydrogenase contributes to pathogen defense in Arabidopsis. Plant Physiol. 2011;155:1947–59. 21311034 10.1104/pp.110.167163 PMC3091113

[86] Rizzi YS , Cecchini NM, Fabro G et al. Differential control and function of Arabidopsis ProDH1 and ProDH2 genes on infection with biotrophic and necrotrophic pathogens. Mol Plant Pathol. 2017;18:1164–74. 27526663 10.1111/mpp.12470 PMC6638284

[87] Kim D-R , Jeon C-W, Cho G et al. Glutamic acid reshapes the plant microbiota to protect plants against pathogens. Microbiome. 2021;9:244, 1-18. 34930485 10.1186/s40168-021-01186-8 PMC8691028

[88] Manzoor H , Kelloniemi J, Chiltz A et al. Involvement of the glutamate receptor AtGLR3.3 in plant defense signaling and resistance to Hyaloperonospora arabidopsidis. Plant J. 2013;76:466–80. 23952652 10.1111/tpj.12311

[89] Anders S , Huber W. Differential expression analysis for sequence count data. Genome Biol. 2010;11:R106, 1-12. 20979621 10.1186/gb-2010-11-10-r106 PMC3218662

[90] Wang Y , Coleman-Derr D, Chen G et al. OrthoVenn: a web server for genome wide comparison and annotation of orthologous clusters across multiple species. Nucleic Acids Res. 2015;43:W78–84. 25964301 10.1093/nar/gkv487 PMC4489293

[91] eulerr citation info . [cited 21 Jan 2021]. Available: https://cran.r-project.org/web/packages/eulerr/citation.html

[92] Tian T , Liu Y, Yan H et al. agriGO v2.0: a GO analysis toolkit for the agricultural community. Nucleic Acids Res. 2017;45:W122–9. 28472432 10.1093/nar/gkx382 PMC5793732

[93] Proost S , Van Bel M, Vaneechoutte D et al. PLAZA 3.0: an access point for plant comparative genomics. Nucleic Acids Res. 2014;43:D974–81. 25324309 10.1093/nar/gku986 PMC4384038

[94] Shannon P , Markiel A, Ozier O et al. Cytoscape: a software environment for integrated models of biomolecular interaction networks. Genome Res. 2003;13:2498–504. 14597658 10.1101/gr.1239303 PMC403769

[95] Goenawan IH , Bryan K, Lynn DJ. DyNet: visualization and analysis of dynamic molecular interaction networks. Bioinformatics. 2016;32:2713–5. 27153624 10.1093/bioinformatics/btw187 PMC5013899

[96] Gómez-Alonso S , Hermosín-Gutiérrez I, García-Romero E. Simultaneous HPLC analysis of biogenic amines, amino acids, and ammonium ion as Aminoenone derivatives in wine and beer samples. J Agric Food Chem. 2007;55:608–13. 17263449 10.1021/jf062820m

[97] Segarra G , Jáuregui O, Casanova E et al. Simultaneous quantitative LC–ESI-MS/MS analyses of salicylic acid and jasmonic acid in crude extracts of Cucumis sativus under biotic stress. Phytochemistry. 2006;67:395–401. 16403544 10.1016/j.phytochem.2005.11.017

