# Supplementary material for: BABA-induced pathogen resistance: a multi-omics analysis of the tomato response reveals a hyper-receptive status involving ethylene
Source: Hortic Res. 2023 Apr 13;10(6):uhad068. doi: 10.1093/hr/uhad068 (PMC10243938; doi:10.1093/hr/uhad068)
Supplement: Web_Material_uhad068 [file web_material_uhad068.zip › Appendix S1.docx]

**S1 Appendix - SUPPLEMENTARY METHODS**

*Protein extraction*

Leaflets were immersed in liquid nitrogen and crushed. 4 g of crushed powder were weighed and mixed with 20 ml of homogenization buffer (50 mM Tris-MES, pH = 8, 20 mM EDTA, 500 mM sucrose, 10 mM DTT, 100 mM PMSF in DMSO). The mixture was homogenized with an Ultraturrax homogenizer at approx. 15,000 rpm at 4 ° C. The samples were further filtered through gauze and 2 protease inhibitor tablets (cOmplete Mini, EDTA-Free, Protease inhibitor cocktail tablets, Roche) was added. Samples were then centrifuged for 25 minutes at 15,557 x rpm and 4 ° C to remove the largest impurities. Supernatants were transferred to 70 ml cuvettes and ultracentrifuged for 35 minutes at 35,000 x rpm and 4°C in a Ti45 rotor on a Beckman Optima centrifuge (Beckman-Coulter). Pellet contained a microsomal membrane fraction, and the supernatant contained a mixture of cytosolic/extracellular proteins. The supernatant was applied to Vivaspin^®^ 6, 3 kDa concentration filters (GE Healthcare) and centrifuged at 10,000 x g and 4 ° C for 2 hours. Supernatants after ultracentrifugation were dialyzed through 3 kDa cut-off dialysis membranes (Cellu · Sep T1 Regenerated cellulose tubular membrane, Orange Scientific) with continuous mixing overnight at 4 ° C in 5 L of 10 mM ammonium acetate solution. The dialyzed samples were then concentrated in Amicon® Ultra-4, 4 mL, 3 kDa centrifugal filters (Merck Millipore Ltd.) at 8,000 x g and 4 ° C to a final volume of 0.5 mL. After concentration, samples final concentration was measured using a BCA protein kit (Sigma-Aldrich).

*Samples fractionation*

Samples were fractionated on an Agilent 1200 HPLC system. Protein samples were injected onto a mixed-bed IEX PolyCATWAX ATM column (column size 200 x 4.6 mm, particle size 5 µm, pore size 1000 Å, PolyLC Inc., Columbia, USA). Mobile phases were 10 mM (A) and 1 M (B) ammonium acetate (pH = 6) with the addition of 5% acetonitrile, respectively. The gradient started with 0% phase B to 10% B at 10 minutes, 50% at 30 minutes and then 100% B at 40 minutes and maintained at 100% until the absorbance returned to baseline. UV absorbance was monitored at 280 nm. The flow rate was set at 1 ml/min and fractions were collected automatically from 3 to 27 minutes. The resulting chromatograms of the triplets of control and BABA-treated samples are shown in Figures 1 and 2. The fractions were then transferred to 50 ml flasks and lyophilized at 25 ° C to remove volatile ammonium acetate on a Christ lyophilizer.


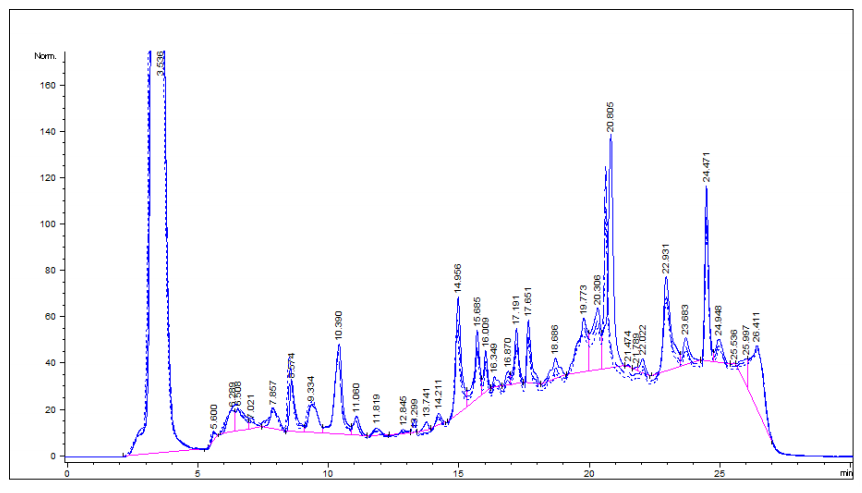


**Figure 1:** **Chromatogram of IEX fractionation of control samples triplicate.** Fractionation of 1 mg protein using a PolyCATWAX ATM column (PolyLC) on an Agilent 1200 at a flow rate of 1 mL/min in an increasing ammonium acetate gradient. Protein detection was performed at 280 nm.


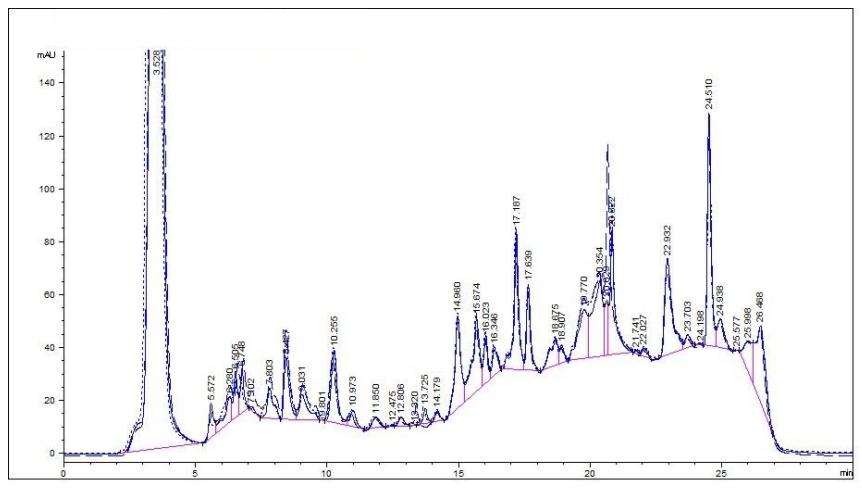


**Figure 2:** **Chromatogram of IEX fractionation of BABA-treated samples triplicate.** Fractionation of 1 mg protein using a PolyCATWAX ATM column (PolyLC) on an Agilent 1200 at a flow rate of 1 mL/min in an increasing ammonium acetate gradient. Protein detection was performed at 280 nm.

*LC-MS/MS analysis of peptides from FASP*

LC-MS/MS analyses of peptide mixtures were done using RSLCnano system connected to Orbitrap Elite hybrid spectrometer (Thermo Fisher Scientific). Before LC separation, tryptic digests were online concentrated and desalted using trapping column (100 μm × 30 mm) filled with 3.5-μm X-Bridge BEH 130 C18 sorbent (Waters). After washing of trapping column with 0.1% FA, the peptides were eluted (flow 300 nl/min) from the trapping column onto an Acclaim Pepmap100 C18 column (3 µm particles, 75 μm × 500 mm; Thermo Fisher Scientific, Waltham, MA, USA) by the following gradient program (mobile phase A: 0.1% FA in water; mobile phase B: 0.1% FA in 80% acetonitrile): the gradient elution started at 1% of mobile phase B and increased from 1% to 56% during the first 100 min (14% in the 30^th^, 30% in the 60^th^ and 56% in 100^th^ min), then increased linearly to 80% of mobile phase B in the next 5 min and remained at this state for the next 15 min. Equilibration of the trapping column and the column was done before sample injection to sample loop. The analytical column outlet was directly connected to the Nanospray Flex Ion Source (Thermo Fisher Scientific, Waltham, MA, USA).

MS data were acquired in a data-dependent strategy selecting up to top 10 precursors based on precursor abundance in the survey scan (350-2000 m/z). The resolution of the survey scan was 60 000 (400 m/z) with a target value of 1×10^6^ ions, one microscan and maximum injection time of 200 ms. HCD MS/MS spectra were acquired with a target value of 50 000 and a resolution of 15 000 (400 m/z). The maximum injection time for MS/MS was 500 ms. Dynamic exclusion was enabled for 45 s after one MS/MS spectra acquisition and early expiration was disabled. The isolation window for MS/MS fragmentation was set to 2 m/z.

The analysis of the mass spectrometric RAW data files was carried out using the Proteome Discoverer software (Thermo Fisher Scientific; version 1.4) with in-house Mascot (Matrixscience, London, UK; version 2.4.1) and Sequest search engines utilisation. MS/MS ion searches were done against protein database downloaded from <ftp://ftp.solgenomics.net/tomato_genome/annotation/ITAG2.4_release/ITAG2.4_proteins_full_desc.fasta> (containing 34,725 sequences) with additional sequences from the cRAP database (downloaded from http://www.thegpm.org/crap/). Mass tolerance for peptides and MS/MS fragments were 10 ppm and 0.05 Da, respectively. Oxidation of methionine and deamidation (N, Q) as optional modification, carbamidomethylation of C as fixed modification and two enzymes (trypsin) miss cleavages were set for all searches. Percolator was used for post-processing of Mascot and Sequest search results. Peptides with false discovery rate (FDR; q-value) < 1%, rank 1 and with at least 6 amino acids were considered. Label-free quantification using protein area calculation in Proteome Discoverer was used (“top 3 protein quantification”) [1].

*LC-MS analyses of salicylic acid, jasmonic acid and jasmonic acid-isoleucine*

Qualitative and quantitative LC-MS analyses were performed with a TOF mass spectrometer (Agilent Technologies) equipped with a dual ESI ion source operated in negative ionization mode. The TOF-MS was coupled online to a high-performance liquid chromatography (LC) system (Agilent Technologies). The drying gas temperature was set to 325°C, the drying gas flow to 10 l/min, the nebulizer pressure to 35 psi, the VCap voltage to 4000 V, the fragmentor voltage to 200 V, the skimmer voltage to 65 V, and the Octopole RF Peak voltage to 750 V. Chromatographic separation was performed on a Zorbax Extend-C18 column (2.1 × 50 mm, 1.8 µm, Agilent). The LC mobile phases were (A) 0.1% acetic acid in water and (B) methanol. A linear gradient profile with the following proportions (v/v) of solvent B was applied (t (min), %B): (0, 15), (10, 100), (15, 100), with 5 min for re-equilibration. The injection volume was 5 µl and the flow rate was 0.2 ml/min. Retention times under these conditions were 5.5 min for SA, 11.1 min for JA, and 12.9 for ILE-JA. The spectra generated for both compounds using negative ion detection included the deprotonated molecule [M-H] (m/z 137.024 for SA, m/z 209.121 for JA and m/z 322.202 for ILE-JA). Acquisition was done by monitoring the peaks at 137.024 m/z (quantifier) and 93.032 m/z (qualifier) for SA, 209.121 m/z (quantifier) and 210.121 m/z (qualifier) for JA, and 322.202 m/z (quantifier) and 209.117 m/z (qualifier) for JA-ILE.

1. Silva JC, Gorenstein MV, Li G-Z, Vissers JPC, Geromanos SJ. Absolute Quantification of Proteins by LCMSE: A Virtue of Parallel ms Acquisition *S. Mol Cell Proteomics. 2006;5: 144–156. doi:10.1074/mcp.M500230-MCP200
