# Supplementary material for: BABA-induced pathogen resistance: a multi-omics analysis of the tomato response reveals a hyper-receptive status involving ethylene
Source: Hortic Res. 2023 Apr 13;10(6):uhad068. doi: 10.1093/hr/uhad068 (PMC10243938; doi:10.1093/hr/uhad068)
Supplement: Web_Material_uhad068 [file web_material_uhad068.zip › S1_Figure.pptx]

## Slide 1
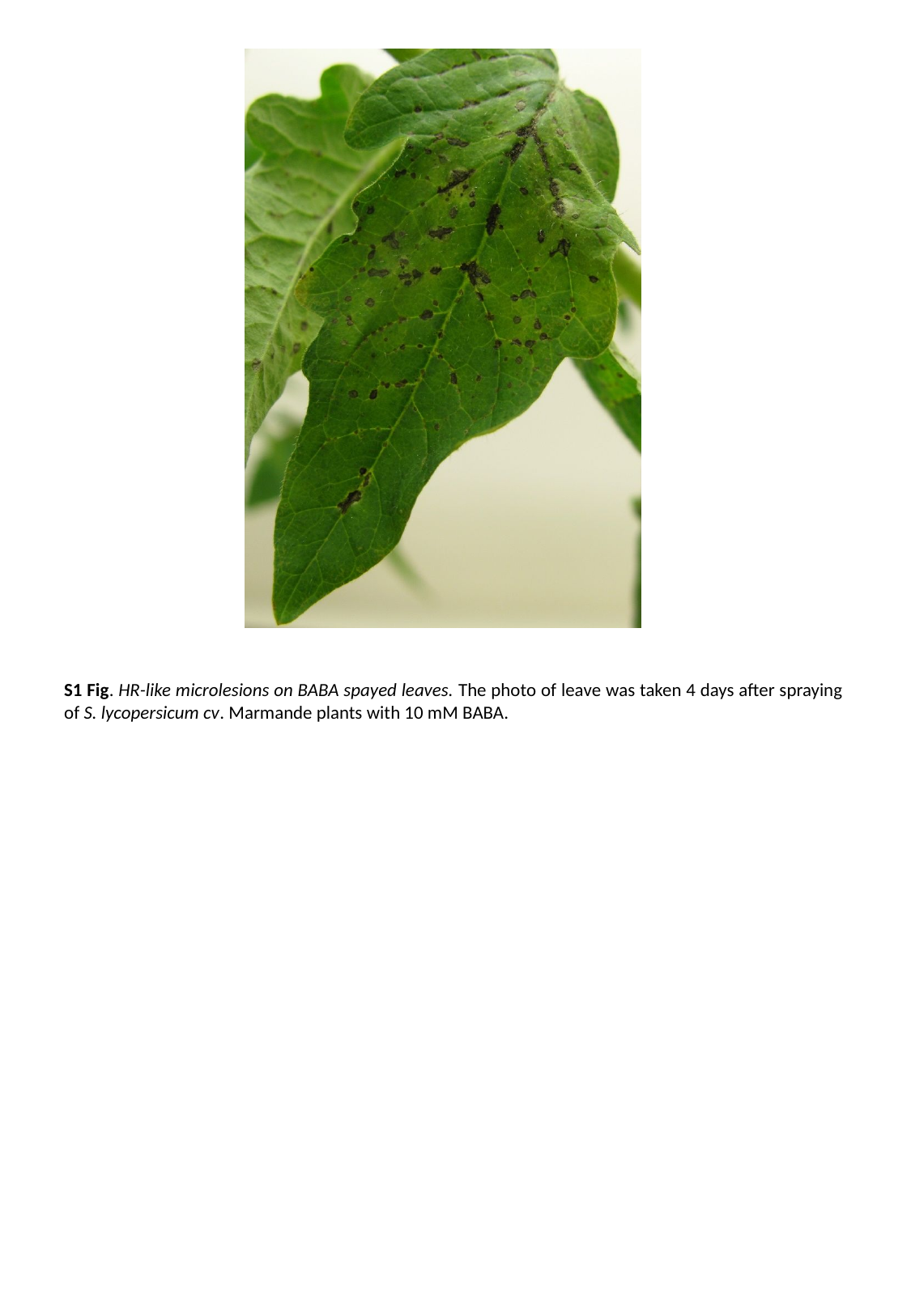

S1 Fig. HR-like microlesions on BABA spayed leaves. The photo of leave was taken 4 days after spraying of S. lycopersicum cv. Marmande plants with 10 mM BABA.
