# Supplementary material for: BABA-induced pathogen resistance: a multi-omics analysis of the tomato response reveals a hyper-receptive status involving ethylene
Source: Hortic Res. 2023 Apr 13;10(6):uhad068. doi: 10.1093/hr/uhad068 (PMC10243938; doi:10.1093/hr/uhad068)
Supplement: Web_Material_uhad068 [file web_material_uhad068.zip › S2_Figure.pptx]

## Slide 1
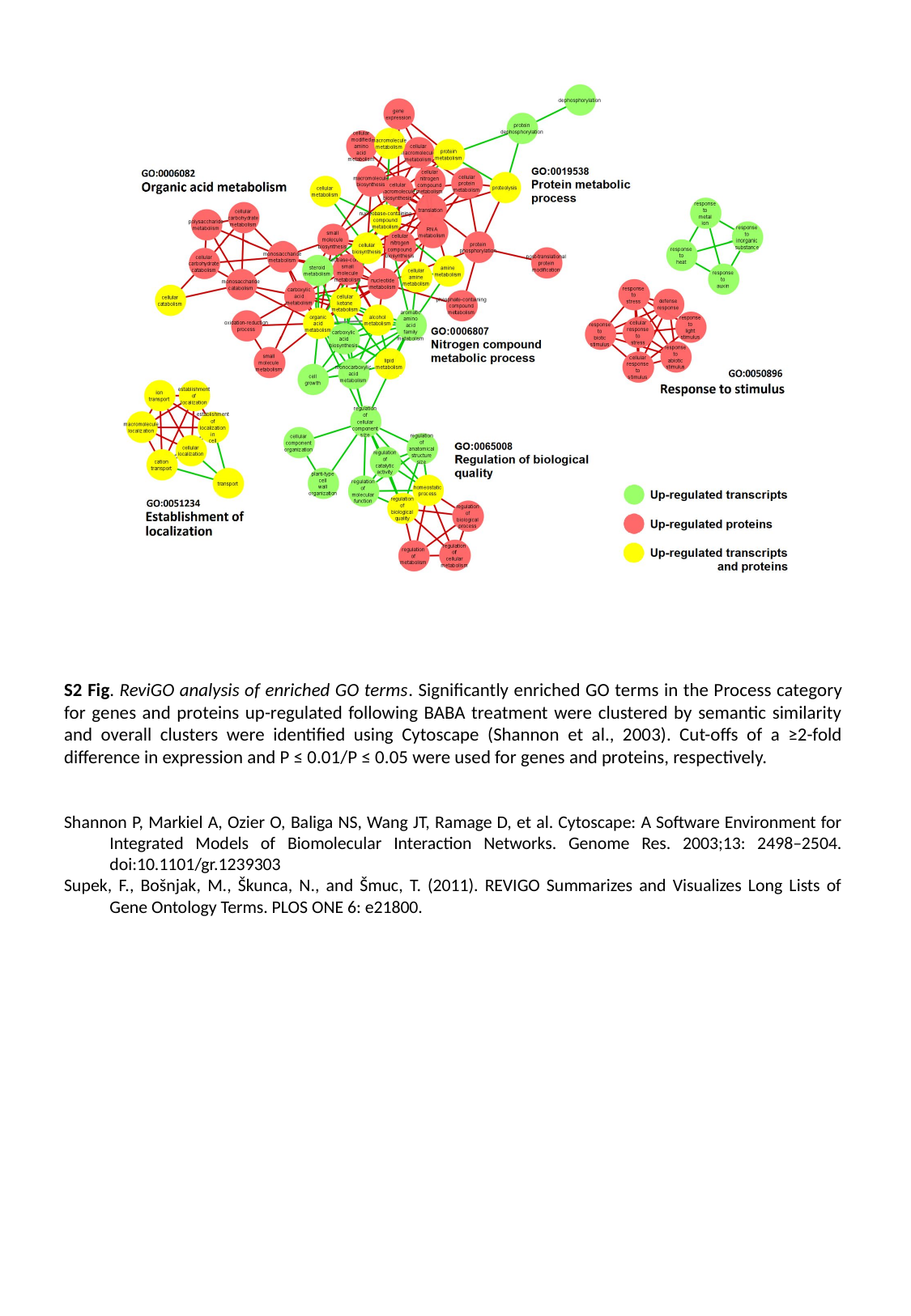

S2 Fig. ReviGO analysis of enriched GO terms. Significantly enriched GO terms in the Process category for genes and proteins up-regulated following BABA treatment were clustered by semantic similarity and overall clusters were identified using Cytoscape (Shannon et al., 2003). Cut-offs of a ≥2-fold difference in expression and P ≤ 0.01/P ≤ 0.05 were used for genes and proteins, respectively.
Shannon P, Markiel A, Ozier O, Baliga NS, Wang JT, Ramage D, et al. Cytoscape: A Software Environment for Integrated Models of Biomolecular Interaction Networks. Genome Res. 2003;13: 2498–2504. doi:10.1101/gr.1239303
Supek, F., Bošnjak, M., Škunca, N., and Šmuc, T. (2011). REVIGO Summarizes and Visualizes Long Lists of Gene Ontology Terms. PLOS ONE 6: e21800.
