# Supplementary material for: BABA-induced pathogen resistance: a multi-omics analysis of the tomato response reveals a hyper-receptive status involving ethylene
Source: Hortic Res. 2023 Apr 13;10(6):uhad068. doi: 10.1093/hr/uhad068 (PMC10243938; doi:10.1093/hr/uhad068)
Supplement: Web_Material_uhad068 [file web_material_uhad068.zip › S3_Figure.pptx]

## Slide 1
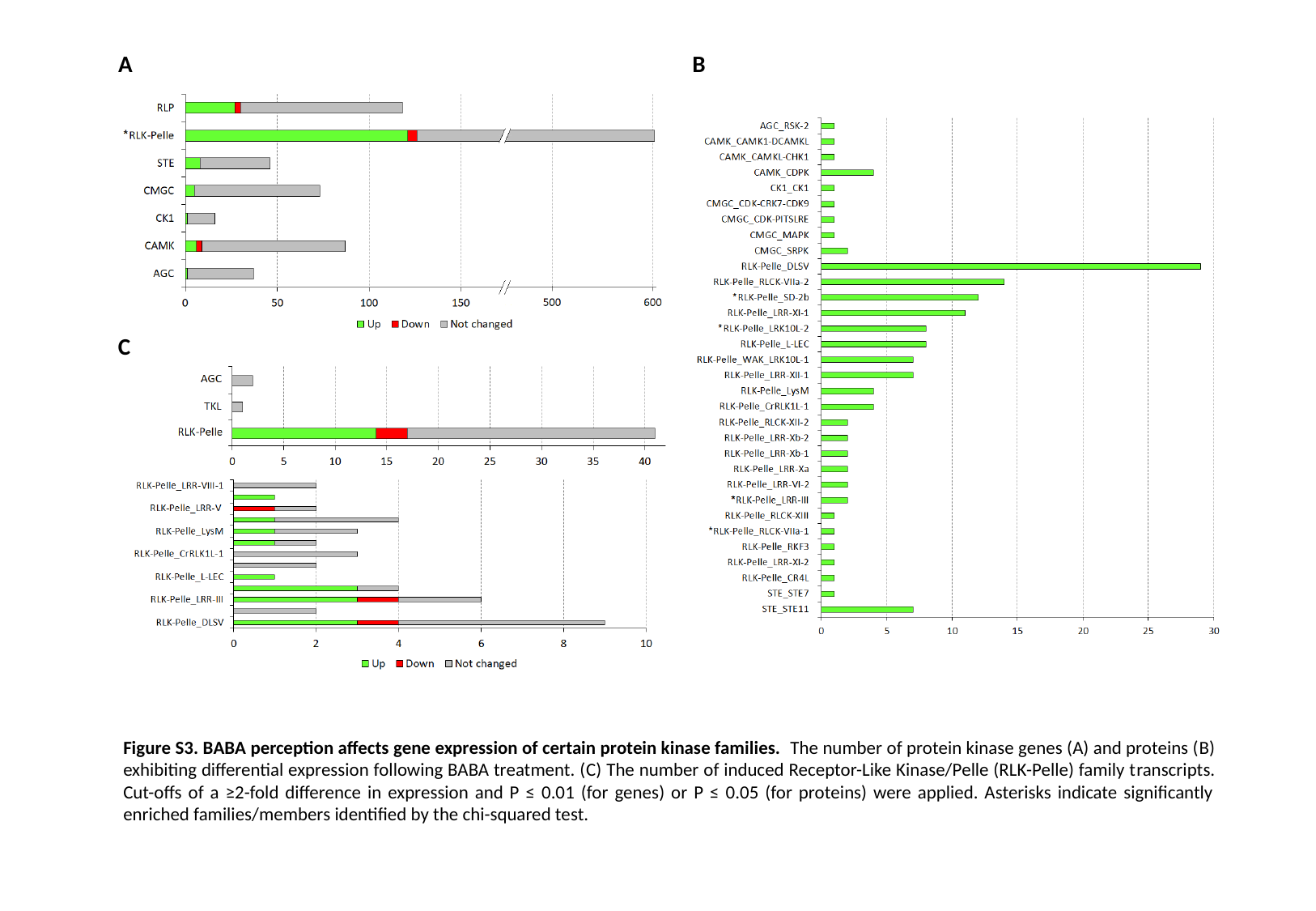

A
B
C
Figure S3. BABA perception affects gene expression of certain protein kinase families. The number of protein kinase genes (A) and proteins (B) exhibiting differential expression following BABA treatment. (C) The number of induced Receptor-Like Kinase/Pelle (RLK-Pelle) family transcripts. Cut-offs of a ≥2-fold difference in expression and P ≤ 0.01 (for genes) or P ≤ 0.05 (for proteins) were applied. Asterisks indicate significantly enriched families/members identified by the chi-squared test.
