# Supplementary material for: BABA-induced pathogen resistance: a multi-omics analysis of the tomato response reveals a hyper-receptive status involving ethylene
Source: Hortic Res. 2023 Apr 13;10(6):uhad068. doi: 10.1093/hr/uhad068 (PMC10243938; doi:10.1093/hr/uhad068)
Supplement: Web_Material_uhad068 [file web_material_uhad068.zip › S4_Figure.pptx]

## Slide 1
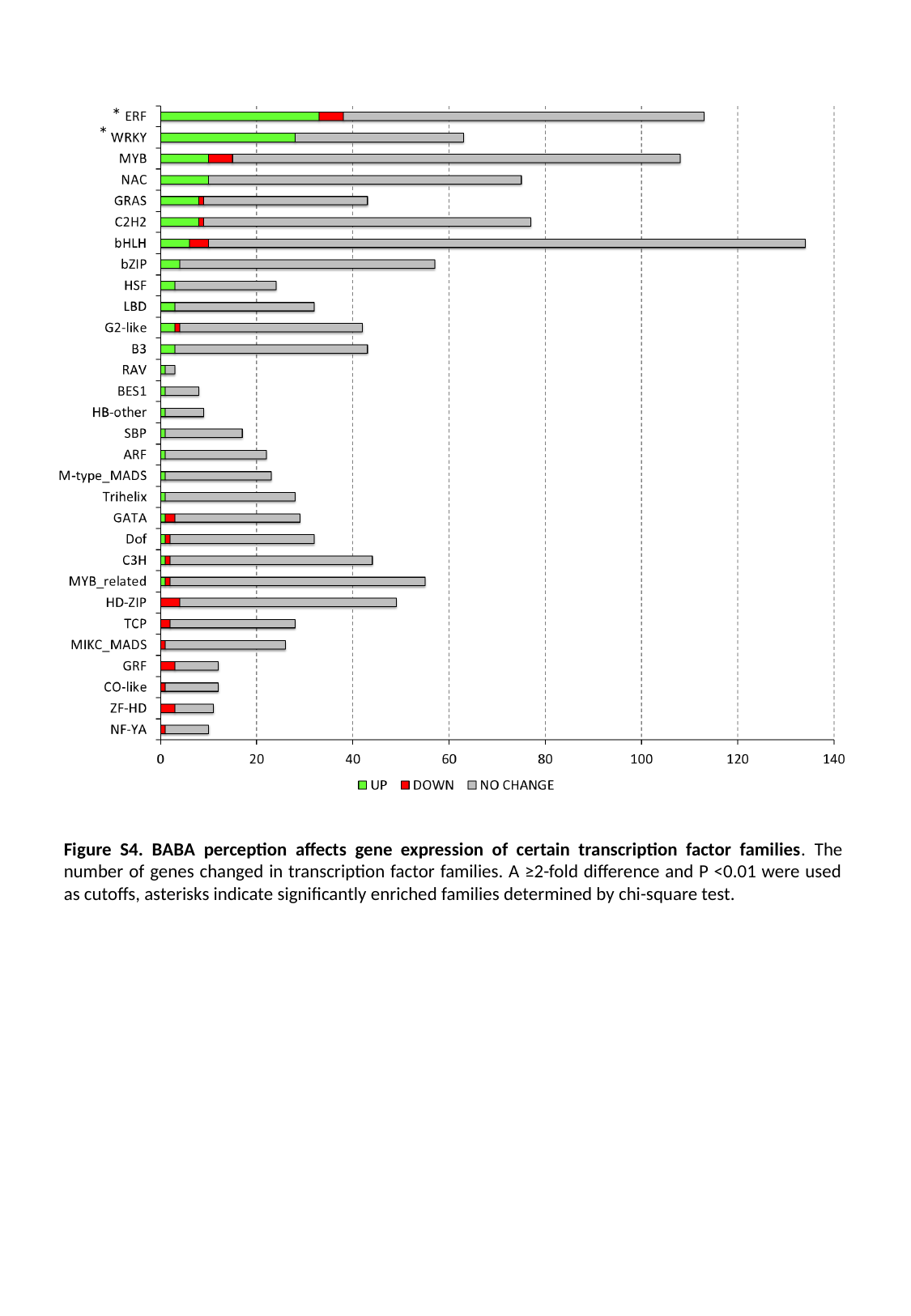

*
*
Figure S4. BABA perception affects gene expression of certain transcription factor families. The number of genes changed in transcription factor families. A ≥2-fold difference and P <0.01 were used as cutoffs, asterisks indicate significantly enriched families determined by chi-square test.
